# FOLR2^+^ macrophage depletion from intestinal metaplasia to early gastric cancer: single-cell sequencing insight into gastric cancer progression

**DOI:** 10.1186/s13046-024-03245-y

**Published:** 2024-12-19

**Authors:** Yuxin He, Jiayu Wang, Zilin Deng, Huang Feng, Mingzhan Du, Deqing Zhang, Guangbo Zhang, Tongguo Shi, Weichang Chen

**Affiliations:** 1 Jiangsu Institute of Clinical Immunology, The First Affiliated Hospital of Soochow University, 178 East Ganjiang Road, Suzhou, 215000 China; 2 Department of Gastroenterology, The First Affiliated Hospital of Soochow University, Suzhou, 215000 China; 3 Jiangsu Key Laboratory of Clinical Immunology, Soochow University, Suzhou, China; 4 Department of Pathology, The First Affiliated Hospital of Soochow University, Suzhou, China

**Keywords:** Intestinal metaplasia, Early gastric cancer, Malignant transformation, Single-cell RNA sequencing, FOLR2^+^ macrophages, CD8^+^T cells

## Abstract

**Background:**

The immune landscape associated with different subtypes of intestinal metaplasia (IM) and early gastric cancer (EGC) remains unclear. This study aimed to investigate the immune landscape of complete intestinal metaplasia (CIM), incomplete intestinal metaplasia (IIM), and EGC, as well as the underlying mechanisms of EGC progression.

**Methods:**

Gastric biopsy samples were collected from five patients with CIM, six patients with IIM, and four patients with EGC, followed by single-cell RNA sequencing. Multiplex immunohistochemical staining was employed to validate the samples from the aforementioned patients. To elucidate the potential mechanisms involved, in vitro coculture experiments were conducted using FOLR2^+^/FOLR2^−^ macrophages and CD8^+^ T cells. Flow cytometry was utilized to investigate the biological functions of FOLR2^+^ macrophages in the progression of EGC.

**Results:**

Five subpopulations of macrophages were identified in CIM, IIM and EGC samples. FOLR2^+^ macrophages possess antitumor immune potential, and the proportion of FOLR2^+^ macrophage gradually decreased from the CIM stage to the IIM and EGC stages. FOLR2^+^ macrophages were significantly positively correlated with CD8^+^ T cells and activated the cytotoxicity of CD8^+^ T cells via antigen cross-presentation. Additionally, during the progression of EGC, epithelial cells progressively upregulated APP expression, thus inducing necroptosis of FOLR2^+^ macrophages via the APP‒TNFRSF21 axis.

**Conclusions:**

Our work provides an understanding of the potential mechanisms underlying the malignant transformation of IM mediated by FOLR2^+^ macrophages.

**Graphical Abstract:**

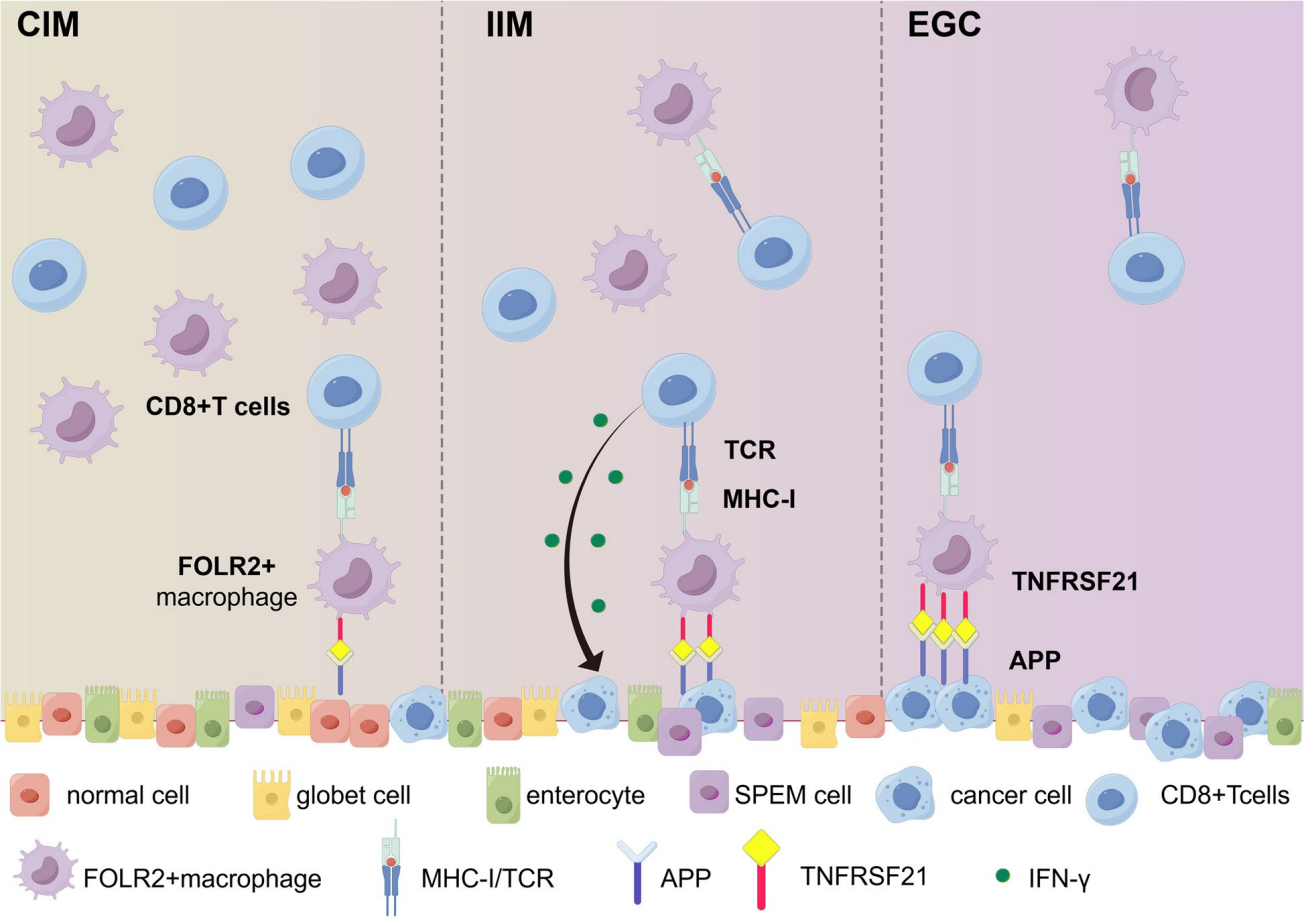

**Supplementary Information:**

The online version contains supplementary material available at 10.1186/s13046-024-03245-y.

## Introduction

Gastric cancer (GC) is still the fourth most common cancer and the fourth leading cause of cancer-related deaths globally [1]. Despite significant progress in GC screening, diagnosis, and treatment, the 5-year overall survival rate for advanced-stage GC remains unfavorable [2]. The carcinogenesis of intestinal-type GC follows a multistep and multistage process known as Correa's model, progressing from normal gastric epithelium through chronic gastritis, chronic atrophic gastritis, intestinal metaplasia (IM), dysplasia, and ultimately to carcinoma [3, 4]. Histologically, gastric intestinal metaplasia can be categorized into two types: complete intestinal metaplasia (CIM) and incomplete gastric intestinal metaplasia (IIM) [5, 6]. Several studies have shown a stronger correlation between IIM and intestinal-type GCs than between CIM and intestinal-type GCs [7–9]. Considering the cost-effectiveness of endoscopic screening, the current GC prevention strategy for most countries involves identifying high-risk populations and subjecting them to endoscopic surveillance. However, currently utilized markers, such as serological tests for *Helicobacter pylori* and pepsinogen, have limited predictive value for cancer risk [10, 11]. Thus, there is a pressing need for updated biomarkers to identify high-risk populations. Clarifying the precise mechanisms distinguishing CIM from IIM and identifying novel biomarkers will improve the clinical management, surveillance, and therapeutic treatment of IM patients.

Myeloid cells, including macrophages, monocytes, and myeloid-derived suppressor cells, constitute the predominant cell type in solid tumors, comprising nearly half of the total cell population in the tumor microenvironment [12–14]. Among these, tumor-associated macrophages (TAMs) are closely linked to cancer development and progression [12]. Compared with adjacent normal tissue, TAMs exhibit greater diversity within tumor tissues, encompassing a wide array of subsets and states [13, 14]. Advances in single-cell RNA sequencing (scRNA-seq) have revealed various macrophage subgroups, revealing significant functional heterogeneity. However, the phenotypic and functional diversities of TAMs in intestinal-type GC still require investigation.

In the present study, we conducted scRNA-seq analysis on CIM, IIM and EGC tissues to systematically characterize the heterogeneity, differentiation states, and interactions of immune cells and stromal cells during the progression of EGC. Our findings revealed a subset of macrophages expressing FOLR2, which gradually decreased from CIM to IIM to EGC and possessed the characteristics of antitumor immunity. Moreover, FOLR2^+^ macrophages influenced the cytotoxicity of CD8^+^ T cells via antigen cross-presentation. Interestingly, we discovered that the upregulation of APP in epithelial cells induced necroptosis of FOLR2^+^ macrophages by enhancing the interaction with the receptor TNFRSF21. Overall, our results reveal crucial events in the tumorigenesis and progression of EGC, suggesting new biomarkers and targets for the diagnosis, monitoring, and treatment of intestinal-type GC.

## Method

### Human specimens

CIM, IIM, and EGC biopsy samples were obtained from patients undergoing gastroscopy at the First Affiliated Hospital of Soochow University (Suzhou, China). The clinical and pathological information for all patients is detailed in Supplementary Table 1. Peripheral blood samples were collected from healthy volunteers. The experiments involved in human samples were approved by the Institutional Committee of the Hospital Ethics Committee of the First Affiliated Hospital of Soochow University and the informed consent was also obtained from each participant (reference number: 2024–357).

### Alcian blue PAS stain

Alcian blue PAS (AB/PAS) staining (#G1285, Solarbio, Beijing, China) was used to distinguish neutral mucins (magenta) in the stomach from acidic mucins (blue) in the intestinal mucosa. After deparaffinization, the slides were stained with Alcian blue for 15 min, washed in distilled water twice for 5 min, treated with periodic acid for 5 min, and rinsed in distilled water. The subsequent steps included incubation with Schiff’s solution, washing in tap water, differentiation with acid alcohol, and final dehydration, clearing, and mounting. Imaging was performed using the NanoZoomer WS-10 system (Hamamatsu Photonics, Hamamatsu City, Japan).

### High Iron Diamine (HID)/AB Stain

The HID/AB stain was used to differentiate acid mucins into sialomucins and sulfomucins. Sialomucins (blue) are present in the small intestine and colon, whereas sulfomucins (brown) are predominate in the colon. The HID/AB staining was performed according to the manufacturer’s instructions (#BA4120A, Wuhan Jinhong Biotech Development Co., Ltd, China). After deparaffinizing in deionized water, the slides were placed in working solution (mixture of HID-A, HID-B and ferric chloride) at room temperature for 18 ~ 24 h. Then, the sections were rinsed in running tap water, and stained with Alcian blue solution for 15 min. Finally, the slides were dehydrated, cleared and mounted. A NanoZoomer WS-10 imaging system (Hamamatsu Photonics, Hamamatsu City, Japan) was used to scan sections.

### Cell lines

The human gastric mucosal epithelial cell line GES-1 was maintained in Dulbecco’s modified Eagle’s medium (DMEM, #C002, EallBio, Beijing, China). The human gastric cancer (GC) cell line AGS and the colorectal cancer cell line Caco-2 were cultured in RPMI 1640 medium. All culture media were supplemented with 10% FBS and 1% penicillin–streptomycin-amphotericin B solution. The human cell lines were incubated in a humidified atmosphere at 37 °C with 5% CO2. Cells were tested for *Mycoplasma* contamination every 3 months using the Mycoplasma PCR Detection Kit (#C0301S, Beyotime). All cell lines were authenticated by STR assay in April–May 2024 at the latest.

### Intestinal metaplasia (IM) models

Gastric intestinal metaplasia (GIM) is a persistent precancerous lesion in the stomach characterized by the replacement of gastric mucosa with metaplastic mucosa resembling intestinal tissue. The intestinal metaplasia model was constructed as previously described [19]. Briefly, GES-1 cells were starved in DMEM without FBS for 24 h and then incubated with 150 μM chenodeoxycholic acid (CDCA, #474–25-9, MCE) for 24 h. The drug was then washed off and the culture medium was replaced with normal growth medium for recovery. After 48 h, the IM cells were harvested for subsequent experiments. To confirm the successful establishment of IM cell model, we compared the expression levels of intestinal markers (CDX2, KLF4, MUC2, VIL1) in the IM model with those in the positive control Caco-2 cells.

### Preparation of human CD8^+^ T cells

Peripheral blood mononuclear cells (PBMCs) sourced from healthy donors were isolated via density gradient centrifugation using a Lymphocyte Separation Medium Kit (#P8610, Solarbio, Beijing, China). CD8^+^ T cells were subsequently positively selected from the PBMCs employing the Human CD8 Positive Selection Kit II (#17,853, Stemcell, Canada). These selected CD8^+^ T cells were then stimulated with 25 μg/ml CD3/CD28 T -Cell activator (#10,971, Stemcell, Canada) and interleukin-2 (IL-2) (150 U/ml, #200–02, PeproTech, New Jersey, USA). The cells were cultured in RPMI 1640 medium (#A001, EallBio, Beijing, China) supplemented with 10% fetal bovine serum (#10099141c, Gibco, California, USA), β-mercaptoethanol (50 μM, #M3148, Sigma‒Aldrich, St. Louis, USA), MEM containing nonessential amino acids (1:100, #11,140,050, Gibco), and L-glutamine (1:100, #25,030,081, Gibco).

### Human monocyte-derived macrophage (MDM) preparation

Monocytes were isolated from PBMCs through magnetic separation using a Human Monocyte Isolation Kit (#19,359, Stemcell, Canada). The purified monocytes were then plated in 6-well plates at a density of 1 × 10^6^ cells/mL and cultured in macrophage differentiation medium supplemented with M-CSF (50 ng/ml, #78,057, Stemcell, Canada). After 4 days of culture, fresh macrophage differentiation medium was added was replenished. The macrophages intended for the experiments were collected on the sixth day.

### FOLR2^+^ Macrophage Isolation

FOLR2^+^ macrophages were isolated and purified from MDMs through immunomagnetic positive selection using a Human PE Positive Selection Kit (#17,664, Stemcell, Canada). In this process, MDMs were labeled with a PE-conjugated anti-human folate receptor β (FR-β) (BioLegend Cat# 391,703) antibody. The labeled macrophages were subsequently combined with magnetic microbeads and separated using a magnet. Following isolation, both FOLR2^+^ macrophages and FOLR2^−^ macrophages were obtained.

### Macrophage-epithelial cell coculture system

GES-1, IM, and AGS cells were seeded on cell climbing films in 24-well plates and cultured for 6 h. Subsequently, MDMs were introduced for direct coculture with these epithelial cells. After 48 h of coculture, mIHC was performed to evaluate necroptosis in FOLR2^+^ macrophages using FOLR2 and phosphorylated MLKL (p-MLKL) (Abcam Cat# ab187091) as markers. To quantify the degree of colocalization, Pearson's correlation coefficient was calculated using the Colocalization plugin in Fiji software. Moreover, cells from the coculture system were harvested and subjected to flow cytometry analysis to quantify the proportion of FOLR2^+^ macrophages.

### Conditioned media, macrophage and T-cell coculture system

Purified FOLR2^+^ or FOLR2^−^ macrophages were cocultured with conditioned media from GES-1, IM and AGS cells for 6 h. To inhibit the recognition of HLA class I by CD8^+^ T cells, an anti-HLA-A/B/C antibody (1 μg/ml, BioLegend Cat# 311,402) was added to the FOLR2^+^ macrophages. And then macrophages were washed and co-cultured with autologous CD8^+^ T cells at a 1:5 ratio in 12-well plates for 48 h. The Cell Activation Cocktail (#423,304, biolegend, California, USA) was added to the coculture system 4 h prior to the end of the incubation. After 48 h of coculture, CD8^+^ T cells were harvested for flow cytometric analysis (FACS).

### Flow cytometry

The cells were resuspended in 100 µl of binding buffer and preincubated with 5 µl of human Fc receptor blocking solution (#abs9476, Absin, Shanghai, China) for 15 min at 4℃. For cell surface staining, the cells were incubated with appropriate antibodies for 20 min in the dark at 4℃. For intracellular cytokine detection, cells were incubated with Cell Activation Cocktail (#423,304, Biolegend, California, USA) in complete RPMI 1640 at 37℃ for 6 h. Then the cells were fixed and permeabilized with a Fixation/ Permeablization Kit (#554,714, BD Biosciences, New Jersey, USA) for 20 min, and subsequently stained with appropriate antibodies for 20–30 min at 4℃ in the dark. After washing twice, cells were resuspended in the perm/wash buffer for flow cytometric analysis. The specific brands and item numbers of antibodies used are listed in Supplementary Table 2.

### Protein extraction and western blotting assay

All of the cells were lysed in sodium dodecyl sulfate (SDS) lysis buffer supplemented with a protease inhibitor cocktail (catalog #P001, NCM, Suzhou, China). Total protein concentrations were measured via a BCA protein assay kit (#P0010, Beyotime, China). Protein samples were then subjected to SDS-PAGE (#P2012, NCM, China) and subsequently transferred onto a PVDF membrane (#10,600,023, GE Healthcare Life Science, Pittsburgh, USA). Following transfer, the membrane was blocked with 5% BSA (#A8020, Solarbio, Beijing, China) and incubated with primary antibodies at 4 °C overnight. This was followed by incubation with the corresponding HRP-labeled secondary antibodies at room temperature for 45 min. Protein bands were detected via enhanced chemiluminescence (ECL) (#10,100, NCM) and visualized with a Chemi Doc™ MP Imaging System (Bio-Rad, California, USA). The primary antibodies used for western blotting included CDX2 (Proteintech Cat# 60,243–1-Ig), KLF4 (Proteintech Cat# 11,880–1-AP), FOLR2, APP (Proteintech Cat# 25,524–1-AP), and goat anti-mouse (Proteintech Cat#SA00001-1)/rabbit (Proteintech Cat# SA00001-2) IgG. Protein levels were normalized to those of GAPDH (Proteintech Cat# 60,004–1-Ig). The specific brands and item numbers are detailed in Supplementary Table 2.

### Total RNA Isolation and RT-qPCR

Total RNA was extracted via the RNA-Quick Purification Kit (#ES-RN001, YISHAN, China) according to the manufacturer’s instructions. Reverse transcription was performed using the HiScript III RT SuperMix Kit (#R323, Vazyme, China). PCR experiments were conducted on a CFX96 Touch Real-Time PCR system (Bio-Rad, CA, USA). The PCR conditions were as follows: 95 ℃ for 5 min for 1 stage, 95 ℃ for 10 s, and 60 ℃ for 40 cycles. The samples were normalized to GAPDH. The sequences of the primers used in this study are listed in Supplementary Table 2.

### Multiplex Immunohistochemical (mIHC)

Multiplex immunohistochemistry (mIHC) was performed via a four-label five-color multiple fluorescence staining kit (#AFIHC025, AiFang Biological, Changsha, China) following the manufacturer's instructions. After deparaffinization, the slices were treated with citrate antigen repair buffer (pH 6.0) to promote antigen repair, 1% hydrogen peroxide to inhibit endogenous peroxidases, and blocked with 3% bovine serum albumin (BSA) for 30 min. Primary antibody incubation was carried out overnight at 4 ℃. After being washed with PBS, the sections were incubated with the secondary antibody at room temperature for 50 min, followed by incubation with Tyramide signal amplification (TSA) dye at room temperature for 3–15 min. Serial staining cycles were repeated until interesting targets were identified. After being stained with DAPI, the sections were coverslipped with mountant and stored at 4 ℃. Subsequently, the Vectra 3 automated quantitative pathology imaging system (Akoya Biosciences, Marlborough, USA) was subsequently used to scan the sections. The multispectral images were unmixed and analyzed using the inForm Advanced Image Analysis Software. The relevant primary and secondary antibodies are shown in Supplementary Table 2.

#### Single cell RNA sequencing analysis

Detailed methods are provided in the Supplementary methods.

#### Statistical analysis

The data are expressed as mean ± SEM (standard error of the mean) of at least three independent experiments unless indicated. Survival analysis was performed via the Kaplan–Meier method. The Wilcoxon rank sum test with the default parameters was used to identify differentially expressed genes (DEGs). The Kruskal‒Wallis test was performed for multiple comparisons. Pearson correlation analysis was used to calculate the correlation between proportions of cell subtypes. Spearman correlation analysis was used to calculate the correlation between the expression of genes/gene signatures. Statistical significance was determined by *p < 0.05; **p < 0.01; ***p < 10^–3^; ****p < 10^–4^; NS: not significant.

## Results

### Single-cell RNA sequencing reveals various cell types involved in intestinal-type gastric adenocarcinoma carcinogenesis

**Fig. 1:**
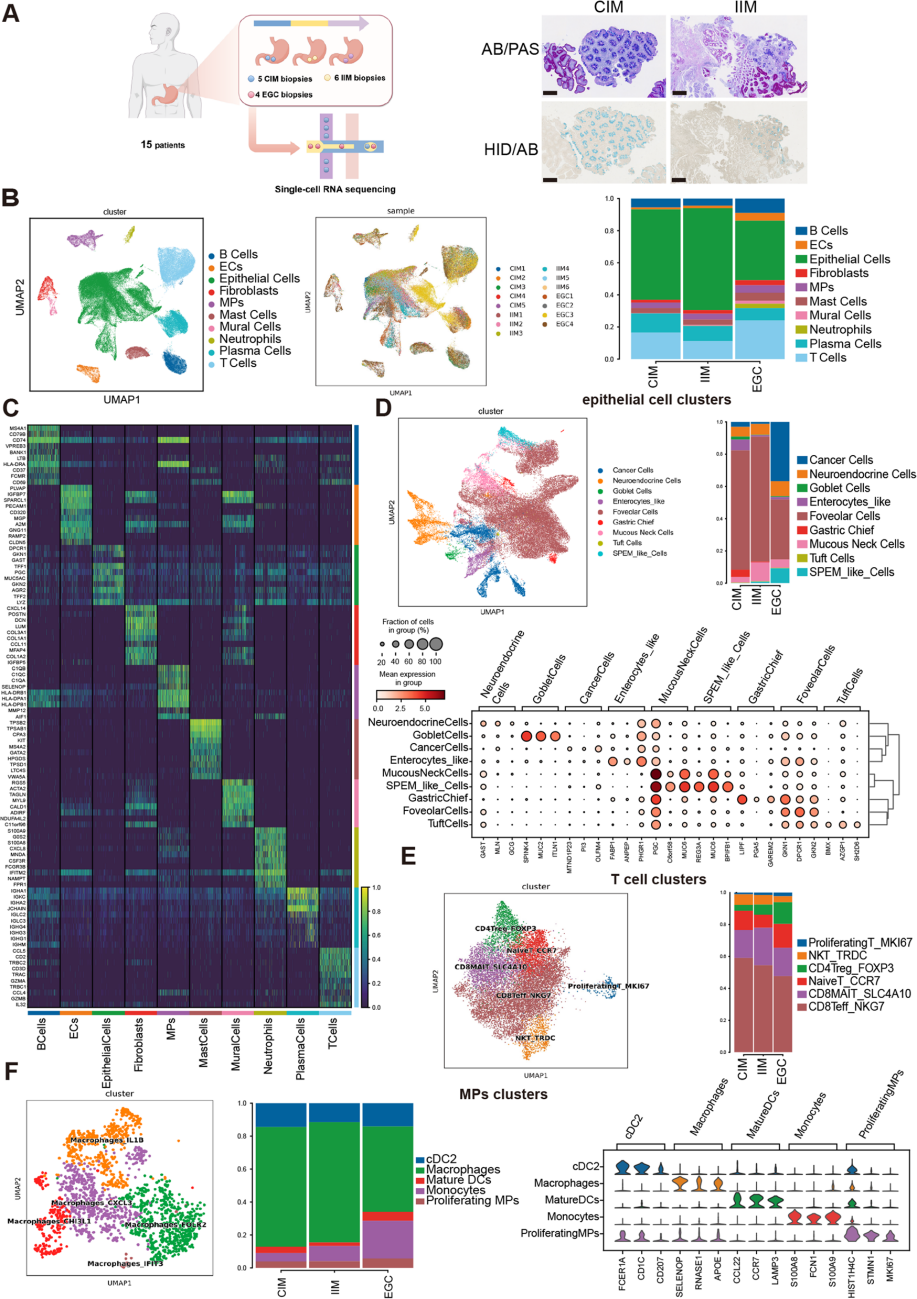
scRNA-seq reveals various cell types involved in intestinal-type GC carcinogenesis. **A** Workflow of sample processing and scRNA-seq (left). AB/PAS and HID/AB staining images of CIM and IIM. Scale bar, 250 μm (right). **B** UMAP plot of single cells annotated by major cell types (left) and samples (middle). The percentages of different cell types in CIM, IIM and EGC samples are shown (right). **C** Heatmap of the DEGs (rows) corresponding to major cell types (columns). **D** UMAP of epithelial cells colored according to inferred cell type (left). Fractions of epithelial cell clusters detected in each group (right). Dot plot of different marker genes of epithelial cell clusters (bottom). **E** UMAP of T cells colored according to inferred cell type (left). Fractions of T-cell clusters detected in each group (right). **F** UMAP of MP clusters colored according to inferred cell type (left). Fractions of MP clusters detected in each group (middle). Violin plots of different marker genes of MP clusters (right)

To unambiguously ascertain whether IIM is closely associated with intestinal-type GC, a *meta*-analysis was conducted with 12 studies (Supplementary Fig. 1A). Compared with CIM, IIM was significantly associated with a greater risk of cancer (Supplementary Fig. 1A). To systematically characterize the single-cell profile of the gastric mucosa during the course of intestinal-type gastric adenocarcinoma carcinogenesis, we performed droplet-based scRNA-seq of five CIM biopsies, six IIM biopsies and four EGC biopsies (Fig. 1A and Supplementary Table 1). After stringent quality control, a total of 96,125 high-quality cells were obtained, including 31,599 cells (32.87%) from CIM tissues, 31,583 cells (32.86%) from IIM tissues, and 32,943 cells (34.27%) from EGC samples. On the basis of graph-based clustering (Fig. 1B) and well-known canonical marker genes (Fig. 1C), these clusters were assigned to ten major cell lineages: epithelial cells (*n* = 50,055, 52.07%), T cells (*n* = 16,682, 17.35%), B cells (6,107 cells, 6.35%), plasma cells (*n* = 9,256, 9.63%), the mononuclear phagocyte system (MPs) (*n* = 3,776, 3.93%), fibroblasts (*n* = 2,312, 2.41%), mast cells (*n* = 3,697, 3.85%), endothelial cells (*n* = 2,456, 2.56%), mural cells (*n* = 815, 0.85%), and neutrophils (*n* = 969, 1.00%) (Fig. 1B, C and Supplementary Fig. 1B; Supplementary Table 1). Regardless of the group or sample, all of the cells were mixed, indicating that the cells clustered due to physiological differences rather than batch effects (Fig. 1B).

To illustrate the diverse cellular ecosystems, the major cell types were further clustered into subtypes. According to the expression patterns of cell type-specific genes, we finally defined nine epithelial cell clusters, including normal gastric clusters (foveolar cells, chief cells, mucous neck cells, neuroendocrine cells), cancer cells, IM cells (goblet cells, enterocytes and tuft cells) and spasmolytic polypeptide-expressing metaplasia (SPEM)-like cells (Fig. 1D). Among them, the copy number variant (CNV) scores of cells obtained via CNV inference were used to distinguish between cancer cells and normal gastric cells (Supplementary Fig. 1C). We observed that < 5% of the cancer cells were in the CIM and IIM stages, whereas the proportion increased to 36.77% in the EGC stage. In contrast, well-differentiated epithelial cell subtypes, such as foveolar cells and chief cells, gradually decreased over the course of the disease (Fig. 1D and Supplementary Table 1). Recently, SPEM cells have been considered precursors of intestinal metaplasia, hyperplasia and tumors [15]. In this study, we established a gene set including *AQP5*, *MUC6*, *TFF2*, *WFDC2*, *CD44v9*, *GKN3*, and *CFTR* to identify SPEM cells [15, 16]. As shown in Supplementary Fig. 1E-F, the mucous neck cell 2 subtype had the highest SPEM score and was defined as a “SPEM-like cell”. Notably, the number of these SPEM-like cells gradually increased from CIM to IIM and EGC (Fig. 1D). These findings were confirmed by multiplex immunofluorescence (Supplementary Fig. 1G). Additionally, the endothelial cells (ECs) were sorted into four subtypes: vascular endothelial cells (VECs), arterial endothelial cells (AECs), capillary endothelial cells (CapECs) and proliferating ECs (Supplementary Fig. 1H and I). The ACE population markedly increased in EGCs (40.46%) compared with IIMs (13.85%) and CIMs (9.83%), whereas CapECs gradually decreased from CIMs to IIMs and EGCs (45.08%, 30.51%, and 8.15%, respectively) (Supplementary Fig. 1H and Supplementary Table 1).

As expected, the proportion of immune cell clusters varied across stages during EGC tumorigenesis (Fig. 1B). Although the proportion of B cells decreased from 5.54% in CIM to 4.44% in IIM, it increased in EGCs (8.97%) (Fig. 1B and Supplementary Fig. 1B). T cells were clustered into sets of CD4Treg_FOXP3, CD8Teff_NKG7, CD8MAIT_SLC4A10, NaiveT_CCR7, NKT_TRDC, and proliferatingT_MKI67 cells (Fig. 1E and Supplementary Fig. 1J). The proportion of CD8Teff_NKG7 in EGCs (47.68%) was substantially lower than that in IIMs (54.48%) and CIMs (59.07%). We observed that the relative percentages of CD4Treg_FOXP3 cells gradually increased from CIM to IIM to EGC tissues (3.66%, 6.33%, and 13.56%, respectively) (Supplementary Table 1).

Next, the heterogeneity and possible functions of myeloid cells in the process of carcinogenesis were explored. We obtained 3,776 myeloid cells and reclustered them into five subsets, including monocytes, proliferating DCs, mature DCs, classic DC2s (cDC2s) and macrophages (Fig. 1F). Overall, from CIM to IIM to EGC, respectively, monocytes (5.11%, 9.23%, and 22.91%) and proliferating MPs (3.94%, 4.07%, and 5.73%) gradually increased, whereas cDC2s (14.47%, 11.51%, and 14.11%) and mature DCs (3.83%, 2.18%, and 5.45%) initially decreased and then increased. The percentage of macrophages, the most abundant cell subset among the MPs, first slightly increased but then decreased (72.66%, 73.02%, and 51.82%) (Supplementary Table 1).

Overall, these results suggest that in intestinal-type GC carcinogenesis, epithelial cells, stromal cells, and immune cell subsets undergo changes.

### Macrophage heterogeneity in intestinal-type gastric adenocarcinoma carcinogenesis

**Fig. 2:**
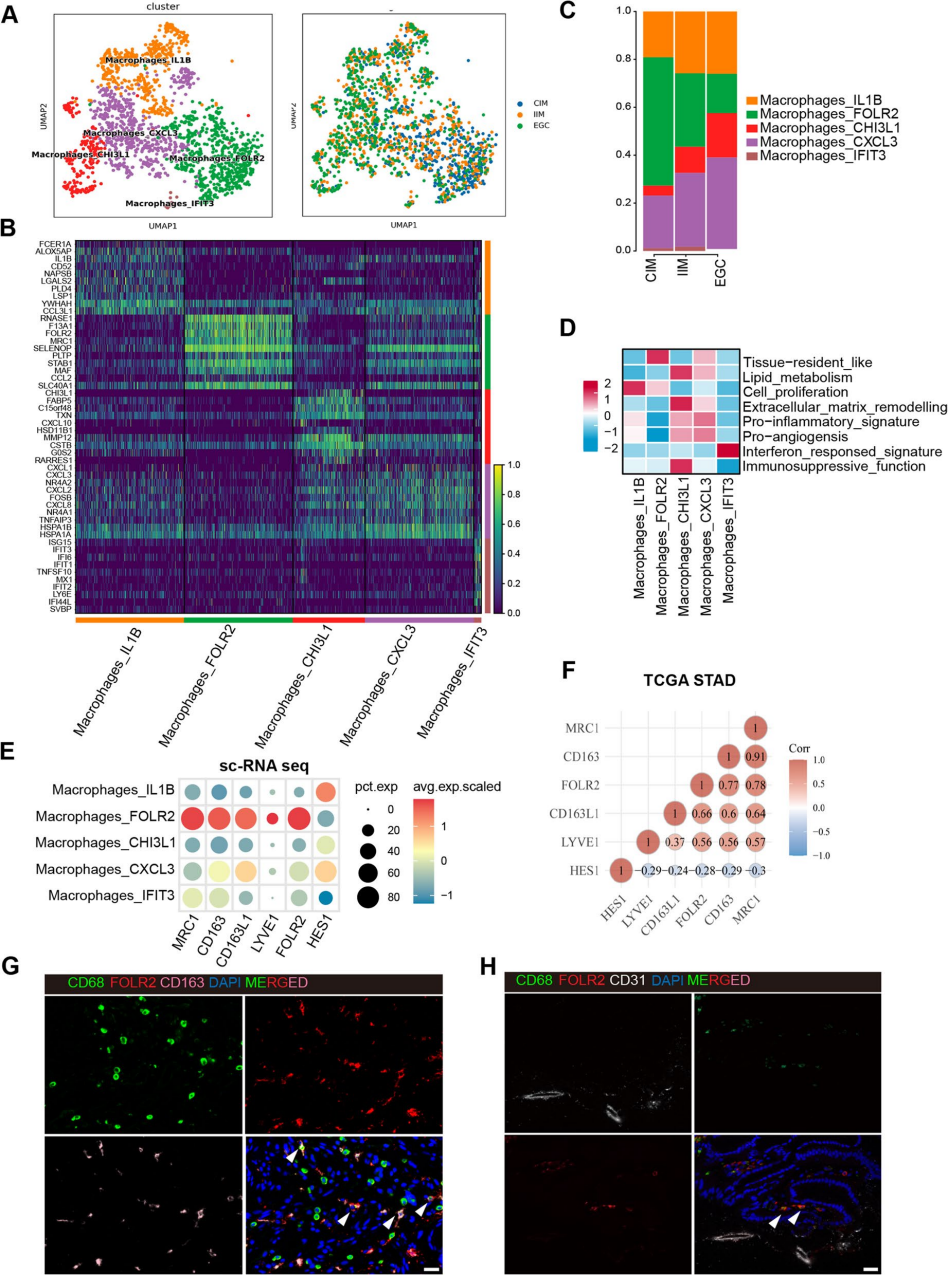
scRNA-seq reveals five subsets of macrophages. **A** UMAP of macrophage clusters colored according to inferred cell type (left) and group (right). **B** Heatmap of the DEGs corresponding to macrophage clusters. **C** Fractions of macrophage clusters detected in CIM, IIM and EGC samples. **D** Heatmap of the macrophage-related gene signature scores in macrophage subsets. **E** Bubble plot of macrophage tissue-resident genes among macrophage clusters. **F** Spearman’s rank correlation between *FOLR2* gene expression and macrophage tissue-resident gene expression in the TCGA-STAD cohort. **G** Representative mIHC images of CD68, FOLR2 and CD163 in CIM tissues. Scale bar, 25 μm. **H** Representative mIHC images of FOLR2 and CD31 in CIM tissues. Scale bar, 25 μm

To decipher the cellular state and function of macrophages in the process of intestinal-type gastric adenocarcinoma carcinogenesis, we reclustered all of the macrophages into five clusters, including macrophage_IL1B, macrophage_CHI3L1, macrophage_CXCL3, macrophage_IFIT3 and macrophage_FOLR2 (Fig. 2A and Fig. 2B). The relative percentages of FOLR2^+^ macrophages gradually decreased from CIM to IIM and EGC tissues. In contrast, the percentages of macrophage_IL1B, macrophage_CHI3L1, macrophage_CXCL3, and macrophage_IFIT3 progressively increased during EGC tumorigenesis (Fig. 2C).

CXCL3^+^ macrophages exhibited upregulated pathways such as the IL-17 signaling pathway and TNF signaling pathway and highly expressed chemokines (*CXCL1, CXCL3* and *CXCL2*) (Fig. 2B, D and Supplementary Fig. 2A, C), suggesting that CXCL3^+^ macrophages may promote immune surveillance and proinflammatory activities. CHI3L1^+^ macrophages were enriched in leukocyte migration, cytokine-mediated signaling pathways, and the response to lipopolysaccharide (Fig. 2D and Supplementary Fig. 2B, D). Additionally, gene set enrichment analysis revealed significant enrichment of protumor gene sets, including ECM remodeling, angiogenesis, lipid metabolism and immunosuppressive functions, in CHI3L1^+^ macrophages (Fig. 2D and Supplementary Table 3), suggesting that CHI3L1^+^ macrophages represent a subset of TAMs with protumor potential. IL-1B^+^ macrophages were significantly enriched in the terms cytoplasmic translation, ribosome, ATP metabolic process and cell proliferation (Fig. 2B, D and Supplementary Fig. 2E, G), suggesting that IL-1B^+^ macrophages are involved in the regulation of tumor growth. The IFIT3^+^ macrophage subset was enriched in interferon-responsive signatures (Fig. 2B, D and Supplementary Fig. 2E, G).

To better understand the functional characteristics of the macrophage subsets, we calculated macrophage function scores for five scRNA-identified macrophage clusters using eight macrophage signature gene sets (Fig. 2D and Supplementary Table 3). In particular, FOLR2^+^ macrophages had the highest tissue-resident_like score among all of the macrophage subtypes (Fig. 2D). These FOLR2^+^ macrophages align with previously described FOLR2^+^ tissue-resident macrophages (TRMs) [17, 18]. Compared with the other macrophage subsets, FOLR2^+^ macrophages expressed higher levels of TRM markers, such as *FOLR2, MRC1, CD163, CD163L1* and *LYVE1*, according to our scRNA-seq data (Fig. 2E). Consistent with the scRNA-seq results, *FOLR2* expression was also positively correlated with TRM-related genes (*CD163, MRC1, CD163L1* and *LYVE1*) in the whole-tumor transcriptome from the TCGA STAD database (Fig. 2F). Moreover, the mIHC results revealed the colocalization of CD68, CD163 (a TRM marker gene) and FOLR2 (Fig. 2G). Notably, we observed that FOLR2^+^ CD68^+^ macrophages resided in close spatial proximity to CD31^+^ vessels in CIM tissues (Fig. 2H), suggesting that FOLR2^+^ macrophages are perivascular TRMs associated with healthy gastric mucosa.

### FOLR2^+^ macrophages exhibit antitumor characteristics

**Fig. 3:**
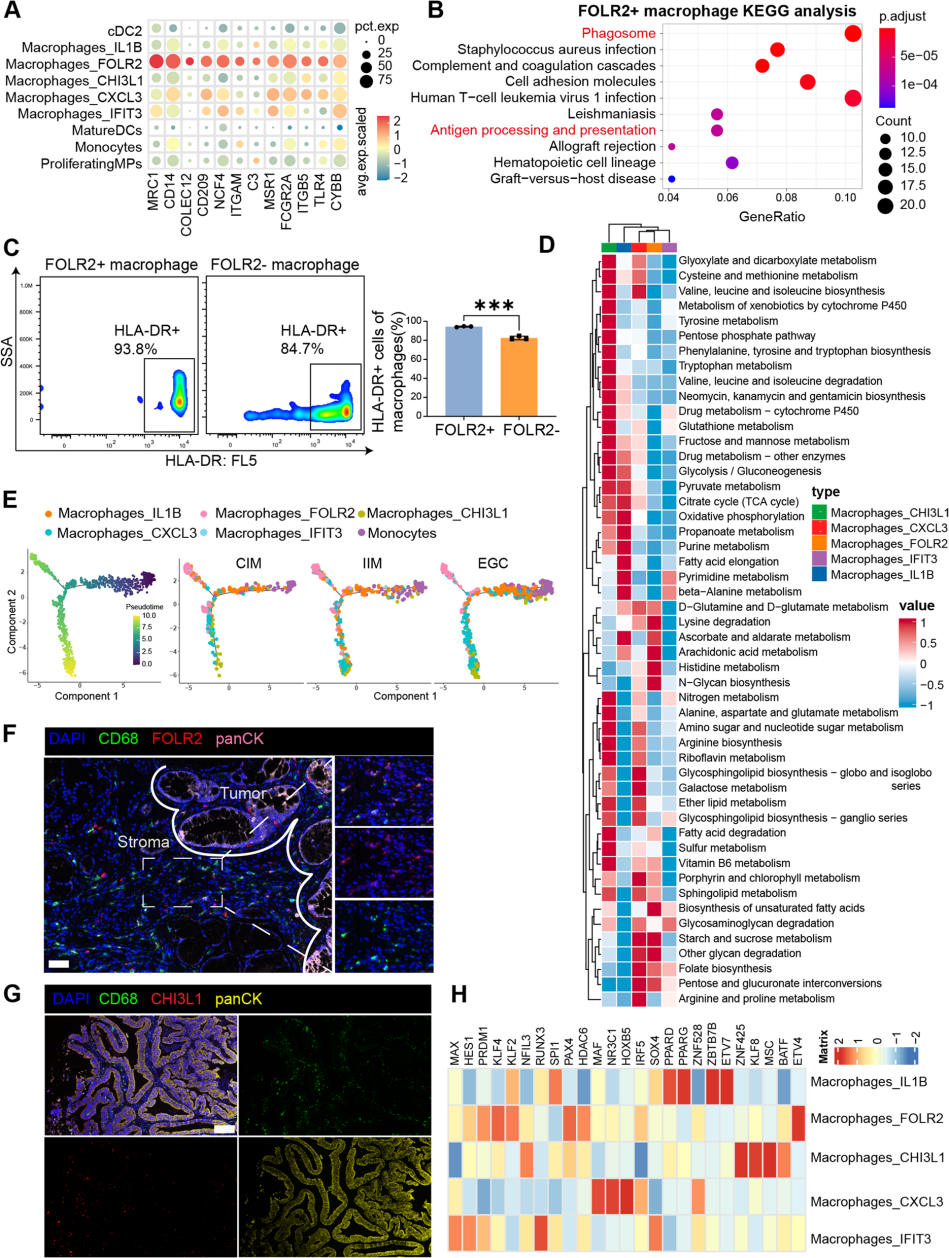
FOLR2^+^ macrophages play crucial roles in antitumor immunity. **A** Bubble plot of phagocytosis-related genes in MP clusters. **B **KEGG analysis of DEGs inFOLR2^+^macrophages.**C** Representative flow cytometry plots of HLA-DR expression in FOLR2^+^ or FOLR2^-^ MDMs from the same healthy donor (*n*=3).** D** Landscape of the metabolic pathways in different macrophage clusters. The color represents the metabolic value.**E** Pseudotime ordering of MP cells into a major trajectory with two bifurcations. The color gradient denotes the pseudotime score (left). Pseudotime trajectory of MP cells presented according to cell clusters for each group (right).**F** Representative mIHC images of FOLR2, CD68 and cytokeratin (CK) in EGC samples. Scale bar, 50 μm.**G** Representative mIHC images of CHI3L1, CD68 and CK in EGC samples. Scale bar, 50 μm. **H** Heatmap of the differentially predicted transcriptional regulons in the five macrophage clusters

To study the biological effects of FOLR2^+^ macrophages, gene enrichment analysis was performed using our scRNA-seq data. As shown in Fig. 3A, FOLR2^+^ macrophages highly expressed notable phagocytic genes. Our analysis revealed that FOLR2^+^ macrophages were characterized by phagosome activation and antigen processing and presentation (Fig. 3B). Moreover, CHI3L1^+^ macrophages highly expressed an immunosuppressive functional gene signature, whereas FOLR2^+^ macrophages were not enriched in immunosuppressive functions (Supplementary Fig. 3A). Given that FOLR2^+^ macrophages have the capacity for antigen presentation, we quantified surface HLA-DR in FOLR2^+^ and FOLR2^−^ macrophages using immunomagnetic sorting of PBMCs from the same healthy donor. Consistent with the scRNA-seq dataset, FACS revealed that FOLR2^+^ macrophages expressed higher levels of HLA-DR than FOLR2^−^ macrophages did (Fig. 3C). Taken together, these results suggest that FOLR2^+^ macrophages are part of the immune context underlying the onset of antitumor immunity.

We identified 652 DEGs between EGC FOLR2^+^ macrophages and IIM FOLR2^+^ macrophages (Supplementary Table 4). GO enrichment analysis revealed that compared with IIM patients, EGC FOLR2^+^ macrophages were enriched in the positive regulation of immune system processes, including antigen processing and presentation, the immune response-activating cell surface receptor signaling pathway, positive regulation of lymphocyte activation, and the MHC protein complex (Supplementary Fig. 3B). To mimic pathological states such as IM and gastric cancer in vitro, we established an intestinal metaplasia (IM) cell model by inducing GES-1 cells with chenodeoxycholic acid (CDCA) exposure [19, 20]. As shown in Supplementary Fig. 3C, CDCA treatment induced a significant increase in classical IM biomarkers, such as CDX2 and KLF4 [21], in IM cells, which were close to those in positive control Caco-2 cells. Moreover, the mRNA levels of *KLF4, MUC2* and *VIL1*, other classical IM biomarkers, were markedly increased in IM cells, similar to the positive control Caco-2 cells (Supplementary Fig. 3D). These results suggest that we successfully constructed a CDCA-induced IM cell model.

Metabolic reprogramming is known to affect immune cells and remold the cellular phenotype and function [22, 23]. To gain further insight into the metabolic activities of the macrophage subsets, we performed scMetabolism [24] on the five macrophage clusters against 85 metabolic pathways in the REACTOME and KEGG databases. Interestingly, the five macrophage subtypes presented distinct metabolic activities (Fig. 3D). In agreement with the findings of a previous study [25], FOLR2^+^ macrophages presented lower metabolic activity (Fig. 3D and Supplementary Fig. 3E), whereas CHI3L1^+^ macrophages were involved in multiple protumor metabolism pathways, such as AA metabolism, OXPHOS, and glycolysis, especially purine metabolism (Fig. 3D and Supplementary Fig. 3E, 3F). These results indicate that these CHI3L1^+^ and CXCL3^+^ macrophage subsets have tumor-promoting potential, whereas FOLR2^+^ macrophages do not play protumor roles from the perspective of the metabolic network.

Next, we performed pseudotime trajectory analysis to explore the developmental state of macrophages from CIM to IIM and EGC. As shown in Fig. 3E, FOLR2^+^ macrophages and CHI3L1^+^ macrophages presented different evolutionary trajectories, with terminal evolutionary statuses. This differentiation process of FOLR2^+^ macrophages was accompanied by the upregulation of *RNASE1*, which enhances the antitumor phenotype of macrophages and the expression of TAM marker genes (*C1QA, C1QB, C1QC, APOE*) (Supplementary Fig. 3G and H).

Previous studies have revealed that macrophages with antitumor activity are often localized at the periphery or surrounding regions of tumors, thereby facilitating immune surveillance functions that identify and eliminate tumor cells [17, 26]. We further performed mIHC staining to validate the presence of the FOLR2^+^ and CHI3L1^+^ macrophage subtypes in the EGC sections. FOLR2^+^ macrophages were predominantly localized in the tumor stroma (Fig. 3F). By contrast, CHI3L1 + macrophages mainly localize close to the tumor nest (Fig. 3G). These results suggest that the localization of macrophages may shape their phenotype and function.

Moreover, we identified candidate regulators of five macrophage subtypes using SCENIC (Fig. 3H). Notably, MAF, NR3C1, HOXB5, IRF5 and ZNF528 were specifically upregulated in FOLR2^+^ macrophages (Fig. 3H). Given that these upregulated genes are associated with terminal macrophage differentiation [27], the phagocytic capacity of macrophages, the activation and proliferation of M1-type macrophages, and set up the environment for a potent T helper type 1 (TH1)-TH17 response [28, 29], FOLR2^+^ macrophages function as antitumor immunity regulators.

To test the association between FOLR2 + macrophage abundance and clinical outcome, we also analyzed whole-tumor transcriptome data from the KM plotter database. Three genes (*C1QA*, *C1QB*, and *C1QC*) define a core macrophage signature shared by the 5 macrophage subtypes identified in our study (Supplementary Fig. 3G-I). We analyzed the gene ratios (FOLR2/C1QA and FOLR2/C1QC) within the bulk transcriptomes of GC samples from the KM plotter database. In accordance with previous reports [17], we found that the highest level of FOLR2^+^ macrophage infiltration was correlated with better overall survival in GC patients (Supplementary Fig. 3J).

### FOLR2^+^ macrophages gradually decreased during EGC tumorigenesis

**Fig. 4:**
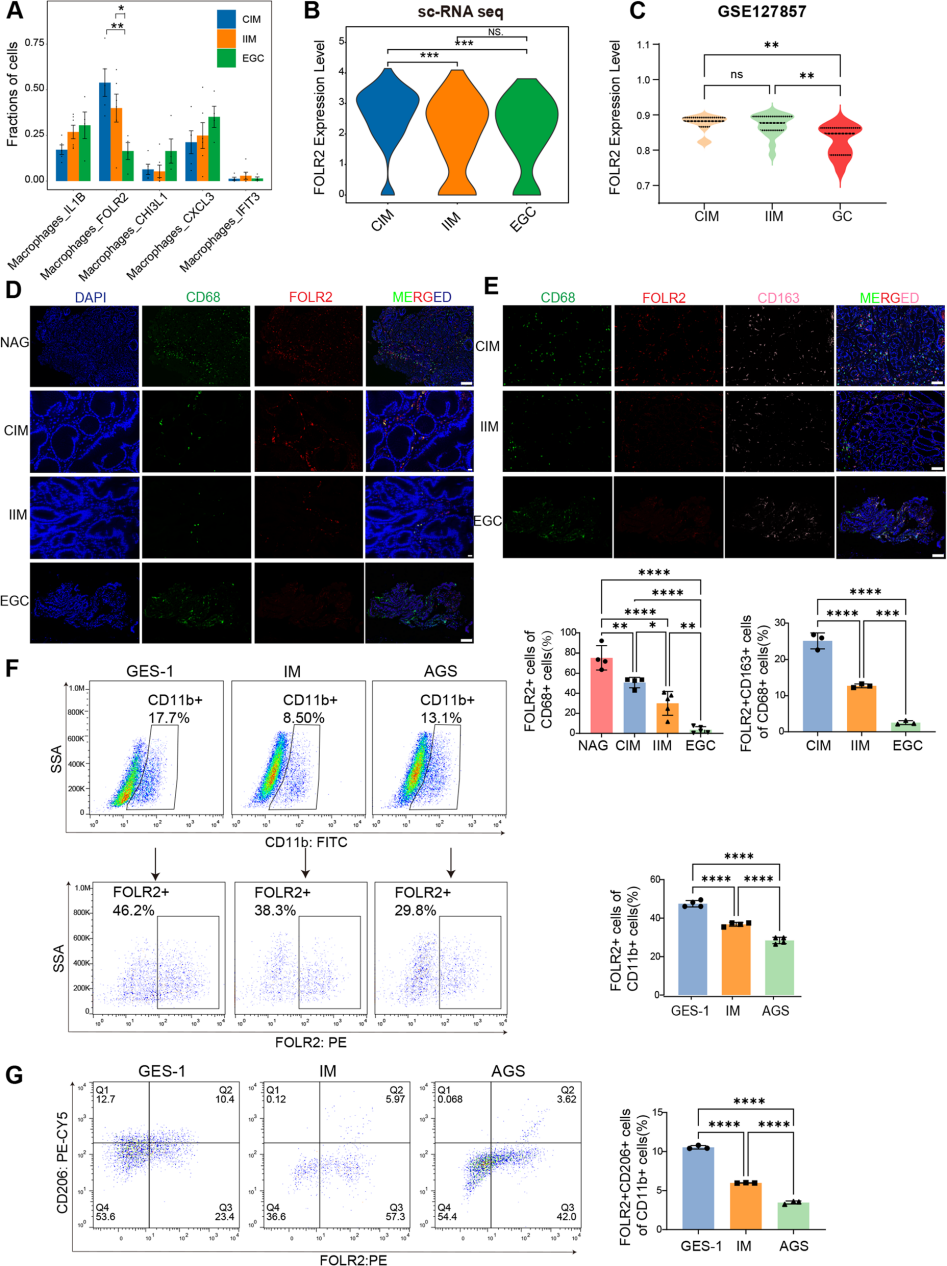
The number of FOLR2^+^ macrophages gradually decrease during EGC tumorigenesis. **A** The proportions of each macrophage cluster in the CIM, IIM and EGC groups from our scRNA-seq data**. B** Violin plots of log-normalized *FOLR2* mRNA expression in FOLR2^+^ macrophages from our scRNA-seq data. **C** Violin plots of log-normalized *FOLR2* mRNA expression in CIM, IIM and EGC tissues from the GSE127857 database. **D** Representative mIHC images of CD68 and FOLR2 in NAG (*n*=4), CIM (*n *=4), IIM (*n*=6) and EGC (*n*=5) tissues. Scale bar, 50 μm. **E **Representative mIHC images of CD68, FOLR2 and CD163 in CIM, IIM and EGC tissues (*n*=3). Scale bar, 50 μm. **F**Representative flow cytometry plots of FOLR2 expression in CD11b^+^MDMs from MDMs cocultured with GES-1, IM and AGS cells (n=4). **G** Representative flow cytometry plots of CD206 and FOLR2 expression in CD11b^+^MDMs from MDMs cocultured with GES-1, IM and AGS cells (*n*=3). The data were analyzed by the Wilcoxon rank-sum test orthe Kruskal‒Wallis test.*p < 0.05; **p < 0.01; ***p < 10^−3^; ****p < 10^−4^; NS: not significant

We further explored the compositional alterations in FOLR2^+^ macrophages across the different disease stages. As shown in Fig. 4A and Supplementary Table 3, FOLR2^+^ macrophages accounted for 53.60% of all macrophages in CIM, and the percentage decreased to 30.68% in IIM and 16.42% in EGC. Compared with those in the IIM and CIM samples, the level of *FOLR2* mRNA was significantly lower in the EGC samples (Fig. 4B). Similar results were observed in the GSE127857 dataset, in which *FOLR2* mRNA expression gradually decreased from CIM to IIM and EGC samples (Fig. 4C). In addition, *FOLR2* mRNA expression was lower in GC patients than in normal controls according to the TCGA database (Supplementary Fig. 4A). mIHC revealed that FOLR2^+^ macrophages (CD68^+^FOLR2^+^) were enriched in nonatrophic chronic gastritis (NAG) and CIM (Fig. 4D). Upon EGC progression, the percentage of FOLR2^+^ macrophages among all CD68^+^ macrophages became relatively low (Fig. 4D). Moreover, mIHC staining revealed that the frequency of CD68^+^CD163^+^FOLR2^+^ macrophages decreased progressively from CIM to IIM to EGC sections (Fig. 4E).

To further investigate the variations in FOLR2^+^ macrophages during IM progression, the IM cell model and GC cells were cocultured with monocyte-derived macrophages (MDMs) to mimic the pathological state in vitro. FACS revealed that the percentage of FOLR2^+^ macrophages gradually decreased among macrophages cocultured with GES-1, IM and AGS cells (Fig. 4F).

Our scRNA-seq results revealed that the *CD163* and *MRC1* expression levels were lower in IIM FOLR2^+^ macrophages than in CIM FOLR2^+^ macrophages, whereas no differences were detected between the IIM and EGC groups (Supplementary Fig. 4B). Additionally, compared with CM from GES-1 cells, CM from IM and AGS cells substantially suppressed the expression of tissue-resident genes, including *CD163L1, LYVE1 and MRC1* in MDMs (Supplementary Fig. 4C). Furthermore, the proportions of FOLR2^+^CD206^+^ macrophages gradually decreased in the MDMs after they were cocultured with GES-1, IM and AGS cells (Fig. 4G). Overall, these data suggest that FOLR2^+^ macrophages significantly decrease during EGC tumorigenesis.

### FOLR2^+^ macrophages fuel CD8^+^ T-cell responses in EGC tumorigenesis

**Fig. 5:**
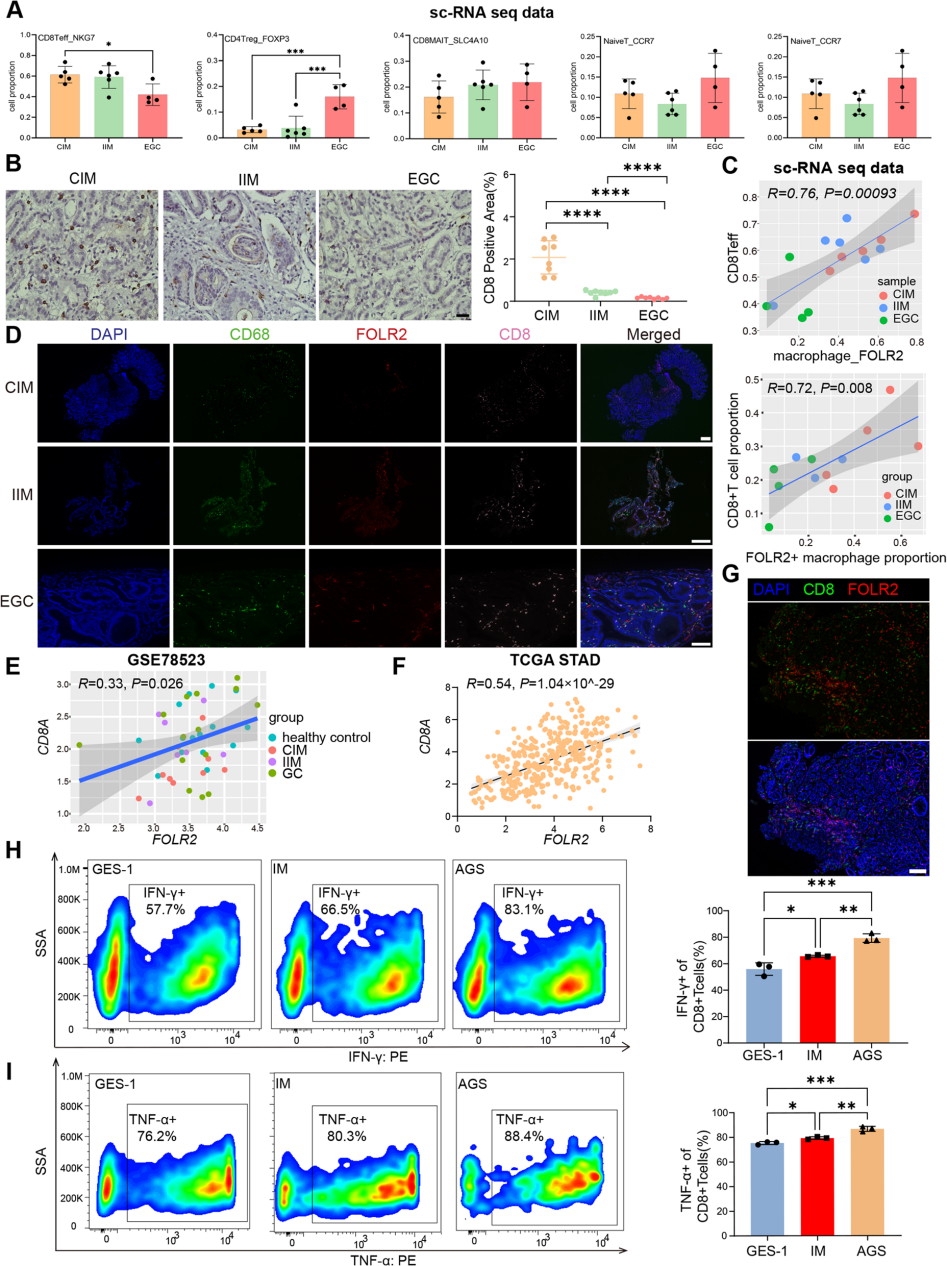
FOLR2^+^ macrophages are positively correlated with CD8^+^ T cells during EGC carcinogenesis. **A** The proportions of each T-cell cluster from our scRNA-seq data. **B** Representative immunohistochemistry images of CD8A in CIM, IIM and EGC tissues (*n*=8). Scale bar, 50 μm. **C** Pearson correlation between FOLR2^+^ macrophages and CD8^+^Teff proportions in CIM, IIM and EGC tissues from our scRNA-seq data. **D** Representative mIHC images of CD68, FOLR2 and CD8 in CIM, IIM and EGC tissues. Scale bar, 100 μm. The Pearson correlation between the proportions of FOLR2^+^macrophages and CD8^+^Teff cells in CIM, IIM and EGC tissues was analyzed. **E** Spearman correlation between *FOLR2* and *CD8A* mRNA expression in healthy control, CIM, IIM and EGC tissues from GSE78523. **F** Spearman correlation between *FOLR2* and *CD8A* mRNA expression and paracancerous normal tissues from the TCGA STAD database. **G** Representative mIHC images of CD8A and FOLR2 expression in NAG tissue. Scale bar, 50 μm. **H** Representative flow cytometry plots of IFN-γ expression in CD8^+^T cells cocultured with FOLR2^+^ macrophages and different epithelial cell supernatants (*n*=3). **I** Representative flow cytometry plots of TNF-α expression in CD8^+^T cells cocultured with FOLR2^+^ macrophages and different epithelial cell supernatants (*n*=3). The data were analyzed by Pearson correlation analysis, Spearman correlation analysis or the Kruskal‒Wallis test. *p < 0.05; **p < 0.01; ***p < 10^−3^; ****p < 10^−4^; NS: not significant

We analyzed the relationship between FOLR2^+^ macrophages and the known antitumor component CD8^+^ T cells in GC carcinogenesis. We observed that the NKG7^+^CD8^+^ Teff proportion gradually decreased from CIM to IIM to EGC samples in both our scRNA-seq data (Fig. 5A) and biopsy samples (Fig. 5B). Notably, our scRNA-seq data revealed that the NKG7^+^CD8^+^ Teff proportion was significantly positively correlated with the FOLR2^+^ macrophage proportion (Fig. 5C and Supplementary Table 5). Moreover, the positive correlation between FOLR2^+^ macrophages (CD68^+^FOLR2^+^) and CD8^+^ T cells was further confirmed by mIHC staining (Fig. 5D). We obtained whole-tumor transcriptomics data from the GSE78523 dataset and observed that the *FOLR2* mRNA level was positively correlated with the *CD8A* mRNA level (Fig. 5E). Moreover, the same correlation was observed in the TCGA stomach adenocarcinoma (STAD) cohort, further confirming this conclusion (Fig. 5F). In addition, on the basis of the TCGA pancancer dataset generated via CIBERSORT, we observed that the *FOLR2* signature was positively correlated with CD8^+^ T cells in BRCA, CESC, CHOL, KICH, LIHC, OV, PAAD, READ, SKCM, THYM, UCEC, UVM, and STAD (Supplementary Fig. 5A). To explore the interactions between FOLR2^+^ macrophages and CD8^+^ T cells, we performed mIHC analysis to observe their spatial relationships in the tissues of NAG patients. FOLR2^+^ macrophages and CD8^+^ T cells were in close proximity in the tissue samples, suggesting that FOLR2^+^ macrophages could directly interact with CD8^+^ T cells (Fig. 5G). We then established a coculture system of FOLR2^±^ macrophages and autologous CD8^+^ T cells with various epithelial cell medium to decode the effect of FOLR2^+^ macrophages on the CD8^+^ T cells during EGC carcinogenesis. Compared with CD8^+^ T cells cocultured with GES-1 supernatants-treated FOLR2^+^ macrophages, CD8^+^ T cells cocultured with FOLR2^+^ macrophages in IM and AGS supernatants showed gradually higher levels of interferon-γ (IFN-γ) and tumor necrosis factor-α (TNF-α) (Fig. 5H and I). In contrast, FOLR2^−^ macrophages inhibited the activation of CD8^+^ T cells during EGC tumorigenesis (Supplementary Fig. 5B and C). These results confirm that FOLR2^+^ macrophages can effectively generate reactive CD8^+^ T cell responses during EGC development.

### FOLR2^+^ macrophages instruct CD8^+^ T-cell expansion and activation by antigen cross-presentation

**Fig. 6:**
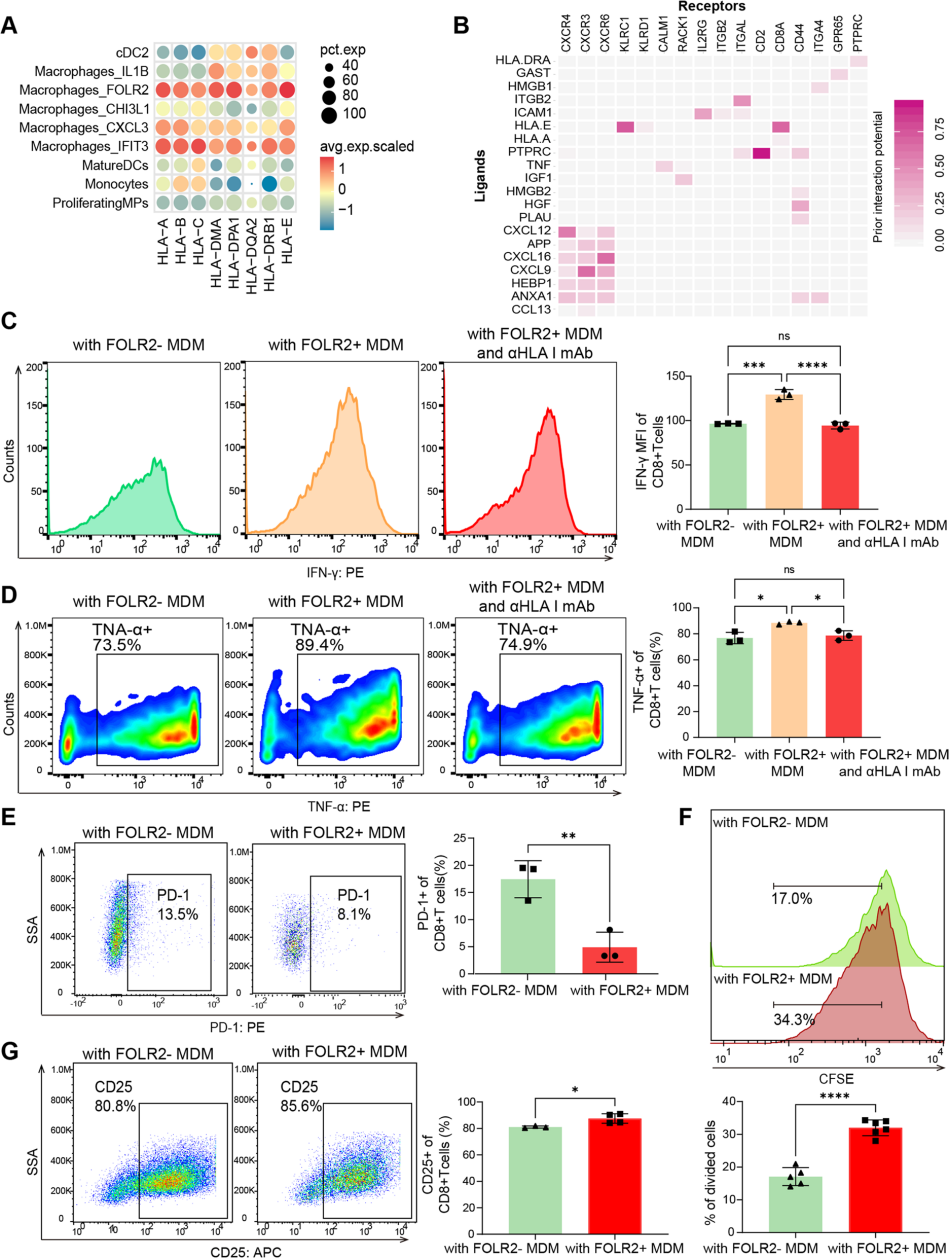
FOLR2 ^+^ macrophages instruct CD8^+^ T-cell expansion and activation by antigen cross-presentation. **A** Bubble plot of MHC molecules in MP clusters. **B** Heatmap of ligand‒receptor interactions between FOLR2^+^ macrophages and CD8^+^ Teff cells via NicheNet. The Y-axis represents the ligands. The X-axis denotes receptors. The color denotes the mean expression level. **C** Representative flow cytometry plots of IFN-γ expression in CD8^+^T cells cocultured with FOLR2^+^ or FOLR2^-^ macrophages in the absence or presence of an anti-HLA class blocking antibody (*n*=3). **D** Representative flow cytometry plots of TNF-α expression in CD8^+^T cells cocultured with FOLR2^+^ or FOLR2^-^ macrophages in the absence or presence of an anti-HLA class blocking antibody (*n*=3). **E** Representative flow cytometry plots of PD-1 expression in CD8^+^ T cells cocultured with FOLR2^+^ macrophages or FOLR2^-^ macrophages (*n*=3). **F** Proliferation capacity of CD8^+^ T cells generated from CFSE-labeled CD8^+^ T cells cocultured with FOLR2^+^ macrophages (*n*=5) and FOLR2^-^ macrophages (*n*=6). **G** Representative flow cytometry plots of CD25 expression in CD8^+^ T cells cocultured with FOLR2^-^ macrophages (*n*=3) or FOLR2^+^ macrophages (*n*=4). The data were analyzed by Student’s t test or the Kruskal‒Wallis test. *p < 0.05; **p < 0.01; ***p < 10^−3^; ****p < 10^−4^; NS: not significant

Macrophages can promote T-cell-mediated antitumor responses by presenting tumor antigens on major histocompatibility complex (MHC) class I and class II to CD8^+^ and CD4^+^ T cells, respectively [30]. GO enrichment analysis revealed that FOLR2^+^ macrophages presented greater antigen processing and presentation functions than the other macrophage subtypes did (Supplementary Fig. 6A), as evidenced by the specific enrichment of MHC class I and class II molecules (Fig. 6A). In addition, FOLR2^+^ macrophages were enriched in positive regulation of leukocyte proliferation (Supplementary Fig. 6A). The receptor‒ligand interaction analysis of FOLR2^+^ macrophages and CD8^+^ Teffs was performed using NicheNet. The results revealed several unique interactions strongly associated with MHC class I cross-presentation, leukocyte recruitment and T-cell activation (ITGB2-ITGAL, ICAM1-IL2RG, PTPRC-CD2, HLA-A-CD8A, CXCL16-CXCR6, and CXCL9-CXCR3) (Fig. 6B). Additionally, *FOLR2* mRNA levels in the bulk transcriptome were positively correlated with genes controlling cytotoxic function in T cells (*NKG7, KLRB1, GZMK, CST7, CCL4, CX3CR1, PRF1,* and *GZMA*) in the TCGA STAD database (Supplementary Fig. 6B). The activation and proliferation of CD8^+^ T lymphocytes require not only MHC class I but also costimulatory molecules (such as CD86). Notably, FOLR2^+^ macrophages highly express MHC class I molecules, as well as the costimulatory molecules CD86 and MHC class II, indicating that they function as specialized antigen-presenting cells (APCs) that primarily promote the expansion and activation of CD8^+^ T cells (Fig. 3C, Fig. 6A and Supplementary Fig. 3A). These results indicate that FOLR2^+^ macrophages are correlated with CD8^+^ T-cell activation and effector functions.

We then established a coculture system of FOLR2^±^ macrophages and autologous CD8^+^ T cells with AGS cell medium to determine the effects of FOLR2^+^ macrophages on CD8^+^ T cells (Supplementary Fig. 6C and D). Compared with CD8^+^ T cells cocultured with FOLR2^−^ macrophages, CD8^+^ T cells cocultured with FOLR2^+^ macrophages showed higher levels of IFN-γ and TNF-α. An anti-HLA-A/B/C blocking antibody reversed this effect (Fig. 6C and Fig. 6D). Moreover, we investigated whether FOLR2^+^ macrophages could modulate the exhaustion of CD8^+^ T cells. As shown in Fig. 6E and Supplementary Fig. 6E, CD8^+^ T cells cocultured with FOLR2^+^ macrophages exhibited lower levels of PD-1 and Tim-3 than CD8^+^ T cells cocultured with FOLR2^−^ macrophages. In addition, FOLR2^+^ macrophages induced CD8^+^ T-cell expansion (Fig. 6F). Moreover, FOLR2^+^ macrophages promote the differentiation of CD8^+^ T cell (upregulation of CD25) (Fig. 6G). These results confirm that FOLR2^+^ macrophages are able to cross-present antigens and activate CD8^+^ T cells.

### APP upregulation in epithelial cells promotes necroptosis of FOLR2^+^ macrophages by enhancing interaction with the TNFRSF21 receptor

**Fig. 7:**
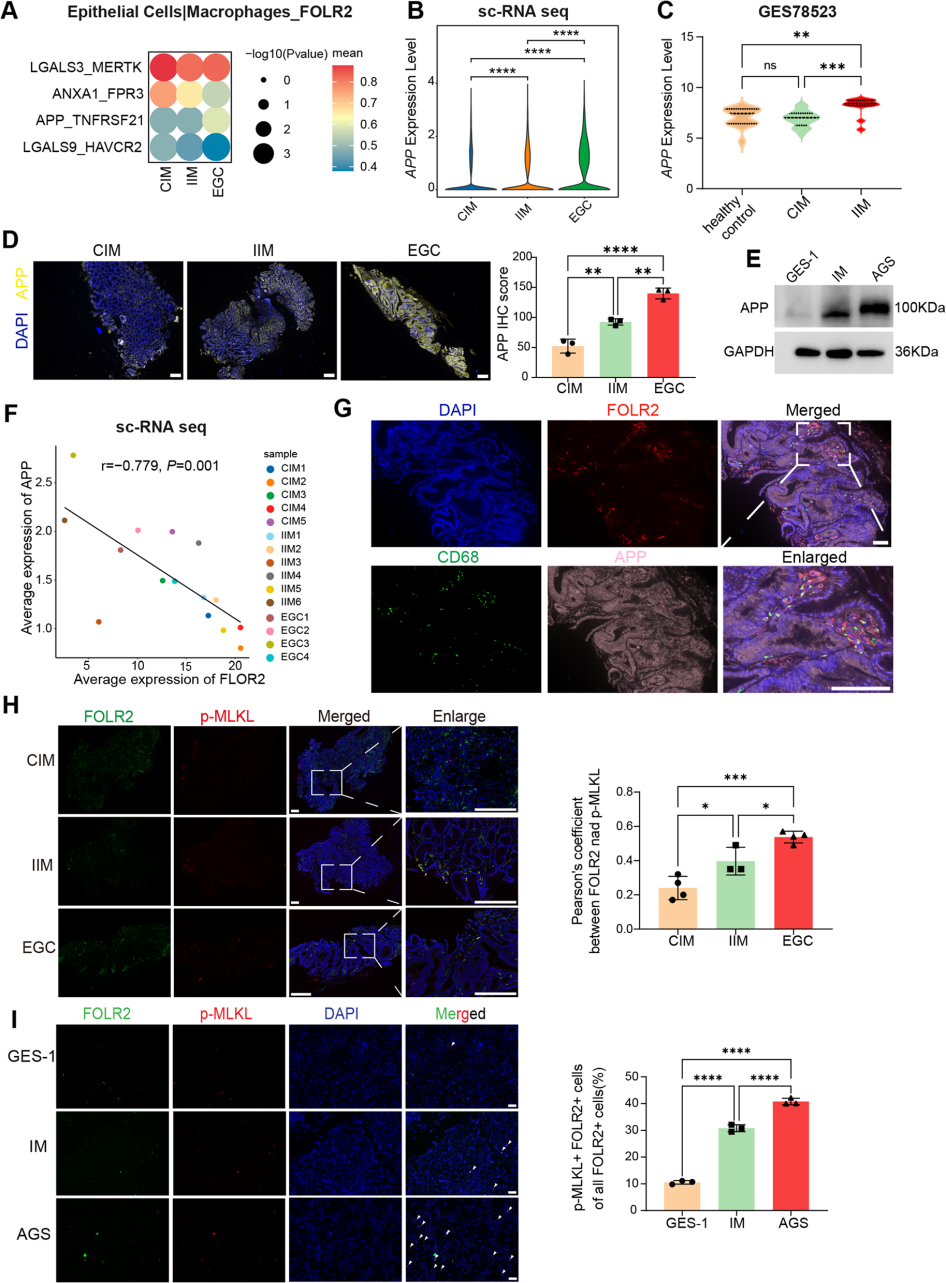
APP upregulation in epithelial cells promotes necroptosis of FOLR2^+^ macrophages by enhancing the APP‒TNFRSF21 axis. **A** Bubble heatmap of ligand‒receptor interactions between epithelial cells and FOLR2^+^macrophages via CellPhoneDB. The Y-axis represents ligand‒receptor pairs. The X-axis denotes groups. The color of the circle denotes the mean expression level. **B** Violin plots of log-normalized *APP *mRNA expression in epithelial cells from our scRNA-seq data. **C** Violin plots of log-normalized *APP* mRNA expression in healthy control, CIM and IIM tissues from the GSE78523. **D** Representative mIHC images of APP expression in CIM, IIM and EGC tissues (*n*=3). Scale bar, 200 μm. **E** Western blot analysis of APP protein levels in GES-1, IM and AGS cells. **F** Spearman correlation between *FOLR2* expression in FOLR2^+^ macrophages and *APP* expression in epithelial cells from our scRNA-seq data. **G** Representative mIHC images of FOLR2, CD68 and APP in EGC tissues. Scale bar, 50 μm. **H** Representative mIHC images of FOLR2 and p-MLKL expression in CIM (*n*=4), IIM (*n*=3) and EGC (*n*=4) tissues. Scale bar, 200 μm. The quantification of necroptotic FOLR2+ macrophages (FOLR2^+^p-MLKL^+^) from CIM, IIM, and EGC tissues is shown (*n*=3). **I** Representative mIHC images of FOLR2 and p-MLKL expression in macrophages cocultured with GES-1, IM and AGS cells. Scale bar, 200 μm. The quantification of necroptotic FOLR2^+^macrophages is shown. The data were analyzed by Spearman correlation analysis,theWilcoxon rank-sum test or the Kruskal‒Wallis test. *p < 0.05; **p < 0.01; ***p < 10^−3^; ****p < 10^−4^

To gain insights into the signaling events linked to the dysregulation of FOLR2^+^ macrophages during EGC tumorigenesis, we next examined how epithelial cells interact with FOLR2^+^ macrophages. We inferred epithelial cell‒macrophage interactions in CIM, IIM and EGC samples via CellPhoneDB. Interaction analysis revealed that epithelial cells in CIM, IIM and EGC tissues exhibited extensive ligand‒receptor binding with macrophage subsets (Supplementary Fig. 7A). Overall, the interaction intensities of immune-inhibitory ligands, such as LGALS3_MERTK and LGALS9_HAVCR2, were highest in CIM tissues, lower in IIM tissues, and lowest in EGC tissues between epithelial cells and FOLR2^+^ macrophages. In terms of the interaction between epithelial cells and FOLR2^+^ macrophages, the level of MT–RNR2–FPR3 was the highest (Supplementary Fig. 7B). However, high levels of MT–RNR2–FPR3 interactions were detected between epithelial cells and various macrophages (Supplementary Fig. 7B), possibly because of mitochondrial gene contamination during the scRNA-seq analysis process (Supplementary Fig. 7B). CellPhoneDB revealed that the APP‒TNFRSF21 interaction pair was highly expressed specifically in epithelial cell–FOLR2^+^ macrophage interactions (Fig. 7A and Supplementary Fig. 7B). Obviously, the APP‒TNFRSF21 interaction intensity gradually increased during EGC tumorigenesis (Fig. 7A). Therefore, we conducted further studies on the APP‒TNFRSF21 axis.

The *APP* mRNA level gradually increased in the epithelial cells of IIMs and EGCs compared with those of CIMs (Fig. 7B). In agreement with our scRNA-seq data, *APP* gene expression progressively increased from healthy controls to CIM and IIM patients in the GSE78523 (Fig. 7C). mIHC analysis confirmed the significant upregulation of the APP protein in IIM and EGC samples compared with CIM samples (Fig. 7D). In addition, western blot analysis revealed increased levels of APP protein in IM and AGS cells compared with those in GES-1 cells (Fig. 7E). Importantly, correlation analysis revealed a negative correlation between epithelial *APP* expression and *FOLR2* expression in FOLR2^+^ macrophages (Fig. 7F). The results of mIHC analysis revealed that APP^+^ epithelial cells were spatially close to FOLR2^+^ macrophages in EGC tissue samples (Fig. 7G), suggesting that interactions between epithelial cells and FOLR2^+^ macrophages occurred.

Given that the APP‒TNFRSF21 axis mediates tumor cell-induced endothelial necroptosis, we inferred that APP^+^ epithelial cells might induce necroptosis of FOLR2^+^ macrophages via the APP‒TNFRSF21 axis. Our scRNA-seq data confirmed that the *FOLR2* gene was specifically expressed in macrophages (Supplementary Fig. 7D). Therefore, we performed mIHC staining of FOLR2 and p-MLKL, a biomarker of necroptosis, to label necroptotic FOLR2^+^ macrophages (Supplementary Fig. 7E). As shown in Fig. 7H, the proportion of necroptotic FOLR2^+^ macrophages (FOLR2^+^ p-MLKL^+^ macrophages) gradually increased in the tissues from CIM to IIM to EGC. Additionally, we used a coculture system of epithelial cells and macrophages to investigate whether the APP‒TNFRSF21 axis induces necroptosis in FOLR2^+^ macrophages. The results showed that IM and AGS cells induced increased necroptosis in FOLR2^+^ macrophages (Fig. 7I). Moreover, we cocultured MDMs with epithelial cells, and FACS revealed that IM and AGS cells led to a reduction in the number of FOLR2^+^ macrophages, and this reduction was reversed by NSA, an inhibitor of necroptosis (Supplementary Fig. 7F). These results suggested that epithelial cells resulted in a decrease in FOLR2^+^ macrophages during EGC tumorigenesis through APP‒TNFRSF21 axis-induced necroptosis of FOLR2^+^ macrophages.

## Discussion

Intestinal-type GC development follows a well-established sequence of histological changes, starting from the normal gastric epithelium, progressing through the IM, and advancing to dysplasia and ultimately malignancy [9, 31]. IM is pathologically categorized into CIM, also known as Type I IM, and IIM, comprising Type II and Type III IM [5, 6]. In CIM, entire intestinal units are present in the stomach, featuring Paneth cells, crypt base columnar cells, and enterocytes arranged in the typical crypt-to-villus organization of the small intestine. Conversely, IIM has been referred to as “colonic”, which is disorganized and harbors immature goblet cells or glands exhibiting hybrid gastric and intestinal morphologies [8]. A prevalent pattern in IIM is the presence of mixed, immature intestine-like cells in the superficial regions of the glandular unit, alongside basal cells displaying characteristics indicative of SPEM cell differentiation. IIM, characterized by a mixture of gastric and less mature intestinal goblet lineages, suggests a milieu of lineage confusion and is associated with an elevated risk of dysplasia and cancer [15]. Therefore, understanding the molecular mechanisms underlying the development and progression of GC is crucial for developing effective targeted therapies.

Recent research has indicated that various factors contribute to the development and progression of GC [32]. For example, Katherine M. Riera et al. reported that TROP2 is highly expressed in IIM compared with CIM and promotes dysplastic cell growth and behaviors [33]. Additionally, AQP5-expressing SPEM cells were identified in TROP2-positive IIM, indicating a greater risk for the development of GC [34]. Huang et al. utilized scRNA-seq and spatial analysis to reveal changes in tissue ecology and IM lineage diversity, notably identifying a cellular compartment primarily composed of intestinal stem cells associated with EGC [35]. These studies have focused primarily on the functions of epithelial cell lineages during the progression of intestinal-type GC. However, the roles of immune cells and stromal cells remain largely unexplored.

In this study, we performed scRNA-seq on samples of CIM, IIM, and EGC to investigate alterations in various components of the tumor microenvironment. We identified a subset of FOLR2^+^ macrophages that play a crucial role in antitumor immunity during the development of GC.

The traditional macrophage polarization model distinguishes macrophages into two main types: M1-type macrophages, which possess antitumor capabilities, and M2-type macrophages, which generally support tumor progression [36]. However, recent scRNA-seq studies have revealed that in vivo, macrophages exhibit more complex phenotypes and concomitantly express individual M1 and M2 genes [37, 38]. The advancement of scRNA-seq technology has enabled the identification of various tumor-infiltrating macrophage subtypes and their functional roles [37]. For example, TREM2^+^ macrophages have been shown to suppress CD8^+^ T-cell infiltration in hepatocellular carcinoma [39], and SPP1^+^ macrophages interact with FAP^+^ fibroblasts to remodel the extracellular matrix [40]. Our study identified five distinct macrophage clusters through scRNA-seq analysis. Among these clusters, macrophage_IL1B, macrophage_CHI3L1, macrophage_CXCL3 and macrophage_IFIT3 play a significant role in promoting the progression of EGC. In contrast, FOLR2^+^ macrophages are linked to antitumor immunity.

Ramos et al. reported that FOLR2^+^ macrophages, as tissue-resident macrophages, interact with CD8^+^ T cells and contribute to antitumor immunity in breast cancer [17]. Consistent with these findings, our study revealed that FOLR2^+^ macrophages have significant potential in antitumor immunity. These macrophages presented increased antigen presentation capabilities and phagocytic functions. These results indicate the important roles of FOLR2^+^ macrophages in antitumor immunity during EGC carcinogenesis. We further elucidated the molecular mechanisms by which FOLR2^+^ macrophages activate CD8^+^ T cells in GC. Antigen cross-presentation is crucial for initiating adaptive immune responses against cancer. Several studies have confirmed that various types of macrophages can contribute to the activation of CD8^+^ T cells via antigen cross-presentation [30, 41]. Our results suggest that FOLR2^+^ macrophages function as specialized APCs and instruct CD8 + T-cell expansion and activation in EGC carcinogenesis.

Given the gradual decrease in FOLR2^+^ macrophages during the progression of EGC, we sought to investigate the underlying reasons for the dysregulation of these macrophages. Hence, we examined the interaction between epithelial cells and FOLR2^+^ macrophages and observed that the intensity of the APP‒TNFRSF21 interaction gradually increased during the tumorigenesis of intestinal-type GC. Strilic et al. revealed that the binding of APP to TNFRSF21 induced endothelial cell necroptosis [42]. Knockdown of APP or blockade of TNFRSF21 results in a significant reduction in endothelial cell necroptosis. In the present study, we provide evidence that the reduced proportion of FOLR2^+^ macrophages is caused by the upregulation of APP in epithelial cells, which enhances the APP‒TNFRSF21 axis and promotes the necroptosis of FOLR2^+^ macrophages. These data suggest that epithelial cells promote necroptosis of FOLR2^+^ macrophages during EGC tumorigenesis through the APP‒TNFRSF21 axis.

One limitation of this study is the small sample size, which may lead to potential confounding statistical biases. Consequently, the results should be further validated in large-scale scRNA-seq cohorts. Moreover, the progression from IM to GC in humans may take several years to decades. There is currently a lack of appropriate animal models that encompass the stages of CIM, IIM, and EGC lesions. Therefore, our experiments did not use animal models and failed to verify the function of FOLR2^+^ macrophages in vivo. Future utilization of patient-derived organoids may aid in validating our results. In addition, the functions of other macrophage subpopulations, immune cells, and stromal cells warrant further investigation. Moreover, the precise molecular mechanisms and regulatory pathways, such as how APP-TNFRSF21 mediates FOLR2^+^ macrophage necroptosis, require in-depth exploration.

## Conclusion

In conclusion, our findings revealed the distinctive immune landscape of EGC and revealed that the number of FOLR2^+^ macrophages gradually decreased from the CIM stage to the IIM stage to the EGC stage. These findings revealed FOLR2^+^ macrophages exhibit antitumor characteristics and shape CD8^+^ T-cell cytotoxicity via antigen cross-presentation. Moreover, epithelial cells induced necroptosis of FOLR2^+^ macrophages via the APP‒TNFRSF21 axis. Consequently, our work provides an understanding of the potential mechanisms underlying IM malignant transformation mediated by FOLR2^+^ macrophages. Moreover, IM patients with low FOLR2^+^ macrophage levels may have an elevated risk of GC, indicating that these patients may require more intensive endoscopic monitoring to facilitate early GC detection. This new biomarker may herald a paradigm shift in our approach to gastric cancer screening.

## Supplementary Information

Supplementary Material 1

Supplementary Material 2

Supplementary Material 3

Supplementary Material 4

Supplementary Material 5

Supplementary Material 6

Supplementary Material 7

## Data Availability

The publicly available data from The Cancer Genome Atlas (TCGA) analyzed in this study were obtained from the GDC portal (https://portal.gdc.cancer.gov.) The datasets used and/or analyzed will be available from the corresponding author on reasonable request.

## Abbreviations

IM: Intestinal metaplasia
EGC: Early gastric cancer
CIM: Complete intestinal metaplasia
IIM: Incomplete intestinal metaplasia
GC: Gastric cancer
TAMs: Tumor-associated macrophages
scRNA-seq: Single-cell RNA sequencing
mIHC: Multiplex immunohistochemistry
AB/PAS: Alcian Blue PAS
PBMCs: Peripheral blood mononuclear cells
IL-2: Interleukin-2
p-MLKL: Phosphorylated MLKL
SDS: Sodium dodecyl sulfate
CDCA: Chenodeoxycholic acid
DEGs: Differentially expressed genes
SPEM: Spasmolytic polypeptide-expressing metaplasia
CNV: Copy number variant
ECs: Endothelial cells
VECs: Vascular endothelial cells
AECs: Arterial endothelial cells
CapECs: Capillary endothelial cells
cDC2s: Classic DC2s
TH1: T helper type 1
NAG: Nonatrophic chronic gastritis
MDMs: Monocyte-derived macrophages
APCs: Antigen-presenting cells
IFN-γ: Interferon-γ
TNF-α: Tumor necrosis factor-α

## References

[1] Sung H, Ferlay J, Siegel RL, et al. Global Cancer Statistics 2020: GLOBOCAN Estimates of Incidence and Mortality Worldwide for 36 Cancers in 185 Countries. CA Cancer J Clin. 2021;71:209–49. 33538338 10.3322/caac.21660

[2] Morgan E, Arnold M, Camargo MC, et al. The current and future incidence and mortality of gastric cancer in 185 countries, 2020–40: A population-based modelling study. EClinicalMedicine. 2022;47: 101404. 35497064 10.1016/j.eclinm.2022.101404 PMC9046108

[3] Correa P. Human gastric carcinogenesis: a multistep and multifactorial process–First American Cancer Society Award Lecture on Cancer Epidemiology and Prevention. Cancer Res. 1992;52:6735–40. 1458460

[4] Fann JC, Chiang TH, Yen AM, et al. Personalized risk assessment for dynamic transition of gastric neoplasms. J Biomed Sci. 2018;25:84. 30453970 10.1186/s12929-018-0485-6 PMC6245882

[5] Filipe MI, Munoz N, Matko I, et al. Intestinal metaplasia types and the risk of gastric cancer: a cohort study in Slovenia. Int J Cancer. 1994;57:324–9. 8168991 10.1002/ijc.2910570306

[6] Shah SC, Gawron AJ, Mustafa RA, et al. Histologic Subtyping of Gastric Intestinal Metaplasia: Overview and Considerations for Clinical Practice. Gastroenterology. 2020;158:745–50. 31887261 10.1053/j.gastro.2019.12.004 PMC7302270

[7] Wei N, Zhou M, Lei S, et al. A meta-analysis and systematic review on subtypes of gastric intestinal metaplasia and neoplasia risk. Cancer Cell Int. 2021;21:173. 33731114 10.1186/s12935-021-01869-0 PMC7968216

[8] Correa P, Piazuelo MB, Wilson KT. Pathology of gastric intestinal metaplasia: clinical implications. Am J Gastroenterol. 2010;105:493–8. 20203636 10.1038/ajg.2009.728 PMC2895407

[9] Tong QY, Pang MJ, Hu XH, et al. Gastric intestinal metaplasia: progress and remaining challenges. J Gastroenterol. 2024;59:285–301. 38242996 10.1007/s00535-023-02073-9

[10] Huang RJ, Park S, Shen J, et al. Pepsinogens and Gastrin Demonstrate Low Discrimination for Gastric Precancerous Lesions in a Multi-Ethnic United States Cohort. Clin Gastroenterol Hepatol. 2022;20(950–952): e3. 33434656 10.1016/j.cgh.2021.01.009

[11] den Hollander WJ, Holster IL, den Hoed CM, et al. Surveillance of premalignant gastric lesions: a multicentre prospective cohort study from low incidence regions. Gut. 2019;68:585–93. 29875257 10.1136/gutjnl-2017-314498

[12] Cassetta L, Pollard JW. A timeline of tumour-associated macrophage biology. Nat Rev Cancer. 2023;23:238–57. 36792751 10.1038/s41568-022-00547-1

[13] Ren X, Zhang L, Zhang Y, et al. Insights Gained from Single-Cell Analysis of Immune Cells in the Tumor Microenvironment. Annu Rev Immunol. 2021;39:583–609. 33637019 10.1146/annurev-immunol-110519-071134

[14] Mulder K, Patel AA, Kong WT, et al. Cross-tissue single-cell landscape of human monocytes and macrophages in health and disease. Immunity. 2021;54(1883–1900): e5. 10.1016/j.immuni.2021.07.007 34331874

[15] Goldenring JR, Mills JC. Cellular Plasticity, Reprogramming, and Regeneration: Metaplasia in the Stomach and Beyond. Gastroenterology. 2022;162:415–30. 34728185 10.1053/j.gastro.2021.10.036 PMC8792220

[16] Bockerstett KA, Lewis SA, Noto CN, et al. Single-Cell Transcriptional Analyses Identify Lineage-Specific Epithelial Responses to Inflammation and Metaplastic Development in the Gastric Corpus. Gastroenterology. 2020;159(2116–2129): e4. 32835664 10.1053/j.gastro.2020.08.027 PMC7725914

[17] Nalio Ramos R, Missolo-Koussou Y, Gerber-Ferder Y, et al. Tissue-resident FOLR2(+) macrophages associate with CD8(+) T cell infiltration in human breast cancer. Cell. 2022;185(1189–1207): e25. 10.1016/j.cell.2022.02.021 35325594

[18] Franklin RA, Liao W, Sarkar A, et al. The cellular and molecular origin of tumor-associated macrophages. Science. 2014;344:921–5. 24812208 10.1126/science.1252510 PMC4204732

[19] Lu W, Ni Z, Jiang S, et al. Resveratrol inhibits bile acid-induced gastric intestinal metaplasia via the PI3K/AKT/p-FoxO4 signalling pathway. Phytother Res. 2021;35:1495–507. 33103284 10.1002/ptr.6915 PMC8048559

[20] Li T, Guo H, Li H, et al. MicroRNA-92a-1-5p increases CDX2 by targeting FOXD1 in bile acids-induced gastric intestinal metaplasia. Gut. 2019;68:1751–63. 30635407 10.1136/gutjnl-2017-315318 PMC6839796

[21] Kazumori H, Ishihara S, Rumi MA, et al. Bile acids directly augment caudal related homeobox gene Cdx2 expression in oesophageal keratinocytes in Barrett’s epithelium. Gut. 2006;55:16–25. 16118348 10.1136/gut.2005.066209 PMC1856383

[22] Wang J, Wang Y, Chu Y, et al. Tumor-derived adenosine promotes macrophage proliferation in human hepatocellular carcinoma. J Hepatol. 2021;74:627–37. 33137360 10.1016/j.jhep.2020.10.021

[23] Bian Y, Li W, Kremer DM, et al. Cancer SLC43A2 alters T cell methionine metabolism and histone methylation. Nature. 2020;585:277–82. 32879489 10.1038/s41586-020-2682-1 PMC7486248

[24] Wu Y, Yang S, Ma J, et al. Spatiotemporal Immune Landscape of Colorectal Cancer Liver Metastasis at Single-Cell Level. Cancer Discov. 2022;12:134–53. 34417225 10.1158/2159-8290.CD-21-0316

[25] Li S, Yu J, Huber A, et al. Metabolism drives macrophage heterogeneity in the tumor microenvironment. Cell Rep. 2022;39: 110609. 35385733 10.1016/j.celrep.2022.110609 PMC9052943

[26] Deng Z, Loyher PL, Lazarov T, et al. The nuclear factor ID3 endows macrophages with a potent anti-tumour activity. Nature. 2024;626:864–73. 38326607 10.1038/s41586-023-06950-4 PMC10881399

[27] Lavin Y, Mortha A, Rahman A, et al. Regulation of macrophage development and function in peripheral tissues. Nat Rev Immunol. 2015;15:731–44. 26603899 10.1038/nri3920 PMC4706379

[28] Krausgruber T, Blazek K, Smallie T, et al. IRF5 promotes inflammatory macrophage polarization and TH1-TH17 responses. Nat Immunol. 2011;12:231–8. 21240265 10.1038/ni.1990

[29] Corbin AL, Gomez-Vazquez M, Berthold DL, et al. IRF5 guides monocytes toward an inflammatory CD11c^+^ macrophage phenotype and promotes intestinal inflammation. Sci Immunol. 2020;5(47):eaax6085. 10.1126/sciimmunol.aax6085 PMC7611075 32444476

[30] Muntjewerff EM, Meesters LD, van den Bogaart G. Antigen Cross-Presentation by Macrophages Front Immunol. 2020;11:1276. 32733446 10.3389/fimmu.2020.01276 PMC7360722

[31] Giroux V, Rustgi AK. Metaplasia: tissue injury adaptation and a precursor to the dysplasia-cancer sequence. Nat Rev Cancer. 2017;17:594–604. 28860646 10.1038/nrc.2017.68 PMC5998678

[32] Sugano K, Moss SF, Kuipers EJ. Gastric Intestinal Metaplasia: Real Culprit or Innocent Bystander as a Precancerous Condition for Gastric Cancer? Gastroenterology. 2023;165(1352–1366): e1. 10.1053/j.gastro.2023.08.028 37652306

[33] Riera KM, Jang B, Min J, et al. Trop2 is upregulated in the transition to dysplasia in the metaplastic gastric mucosa. J Pathol. 2020;251:336–47. 32432338 10.1002/path.5469 PMC8010636

[34] Lee SH, Jang B, Min J, et al. Up-regulation of Aquaporin 5 Defines Spasmolytic Polypeptide-Expressing Metaplasia and Progression to Incomplete Intestinal Metaplasia. Cell Mol Gastroenterol Hepatol. 2022;13:199–217. 34455107 10.1016/j.jcmgh.2021.08.017 PMC8593616

[35] Huang KK, Ma H, Chong RHH, et al. Spatiotemporal genomic profiling of intestinal metaplasia reveals clonal dynamics of gastric cancer progression. Cancer Cell. 2023;41(2019–2037): e8. 10.1016/j.ccell.2023.10.004 PMC10729843 37890493

[36] Murray PJ, Allen JE, Biswas SK, et al. Macrophage activation and polarization: nomenclature and experimental guidelines. Immunity. 2014;41:14–20. 25035950 10.1016/j.immuni.2014.06.008 PMC4123412

[37] Cheng S, Li Z, Gao R, et al. A pan-cancer single-cell transcriptional atlas of tumor infiltrating myeloid cells. Cell. 2021;184(792–809): e23. 10.1016/j.cell.2021.01.010 33545035

[38] Zilionis R, Engblom C, Pfirschke C, et al. Single-Cell Transcriptomics of Human and Mouse Lung Cancers Reveals Conserved Myeloid Populations across Individuals and Species. Immunity. 2019;50(1317–1334): e10. 10.1016/j.immuni.2019.03.009 PMC6620049 30979687

[39] Tan J, Fan W, Liu T, et al. TREM2(+) macrophages suppress CD8(+) T-cell infiltration after transarterial chemoembolisation in hepatocellular carcinoma. J Hepatol. 2023;79:126–40. 36889359 10.1016/j.jhep.2023.02.032

[40] Qi J, Sun H, Zhang Y, et al. Single-cell and spatial analysis reveal interaction of FAP(+) fibroblasts and SPP1(+) macrophages in colorectal cancer. Nat Commun. 2022;13:1742. 35365629 10.1038/s41467-022-29366-6 PMC8976074

[41] Ma S, Sun B, Duan S, et al. YTHDF2 orchestrates tumor-associated macrophage reprogramming and controls antitumor immunity through CD8(+) T cells. Nat Immunol. 2023;24:255–66. 36658237 10.1038/s41590-022-01398-6 PMC10150872

[42] Strilic B, Yang L, Albarran-Juarez J, et al. Tumour-cell-induced endothelial cell necroptosis via death receptor 6 promotes metastasis. Nature. 2016;536:215–8. 27487218 10.1038/nature19076

