# Supplementary material for: FOLR2+ macrophage depletion from intestinal metaplasia to early gastric cancer: single-cell sequencing insight into gastric cancer progression
Source: J Exp Clin Cancer Res. 2024 Dec 19;43:326. doi: 10.1186/s13046-024-03245-y (PMC11657096; doi:10.1186/s13046-024-03245-y)
Supplement: Supplementary file 1 — Supplementary Material 1 [file 13046_2024_3245_MOESM1_ESM.docx]

**Supplementary Figures**

**FOLR2^+^ macrophage Depletion From Intestinal Metaplasia To Early Gastric Cancer:**

**Single-cell Sequencing Insight Into Gastric Cancer Progression**

**CONTENT**

[**Supplementary Figure1.** Single-cell transcriptomic analysis reveals various cell types involved in intestinal-type gastric adenocarcinoma carcinogenesis, related to Figure1……………………………………2](#figS1)

[**Supplementary Figure2.**Enrichment analysis of DEGs in five macrophage subtypes, related to Figure2………………………………………………………………………………………………………………………………………3](#figS2)

[**Supplementary Figure3.** FOLR2^+^ macrophages play crucial roles in antitumor immunity, related to Figure3…………………………………………………………………………………………………………………………………4](#figS3)

[**Supplementary Figure4.** FOLR2^+^ macrophages gradually decreased during EGC tumorigenesis，related to Figure4………………………………………………………………………………………………………………………5](#figS4)

[**Supplementary Figure5.** FOLR2^+^ macrophages are positively correlated with CD8^+^ T cells during EGC carcinogenesis, related to Figure5………………………………………………………………………………………6](#figS5)

[**Supplementary Figure6.** FOLR2^+^ macrophages instruct CD8+ T-cell expansion and activation by antigen cross-presentation, related to Figure6………………………………………………………………………………7](#figS6)

[**Supplementary Figure7.** APP upregulation in epithelial cells promotes necroptosis of FOLR2^+^ macrophages by enhancing the APP‒TNFRSF21 axis, related to Figure7……………………………………8](#figS7)

Remarks: Figures were numbers according to the relevant figures it was referenced to in the main manuscript. For example, the content of **Supplementary Figure 2** is relevant to main **Figure 2**.

**
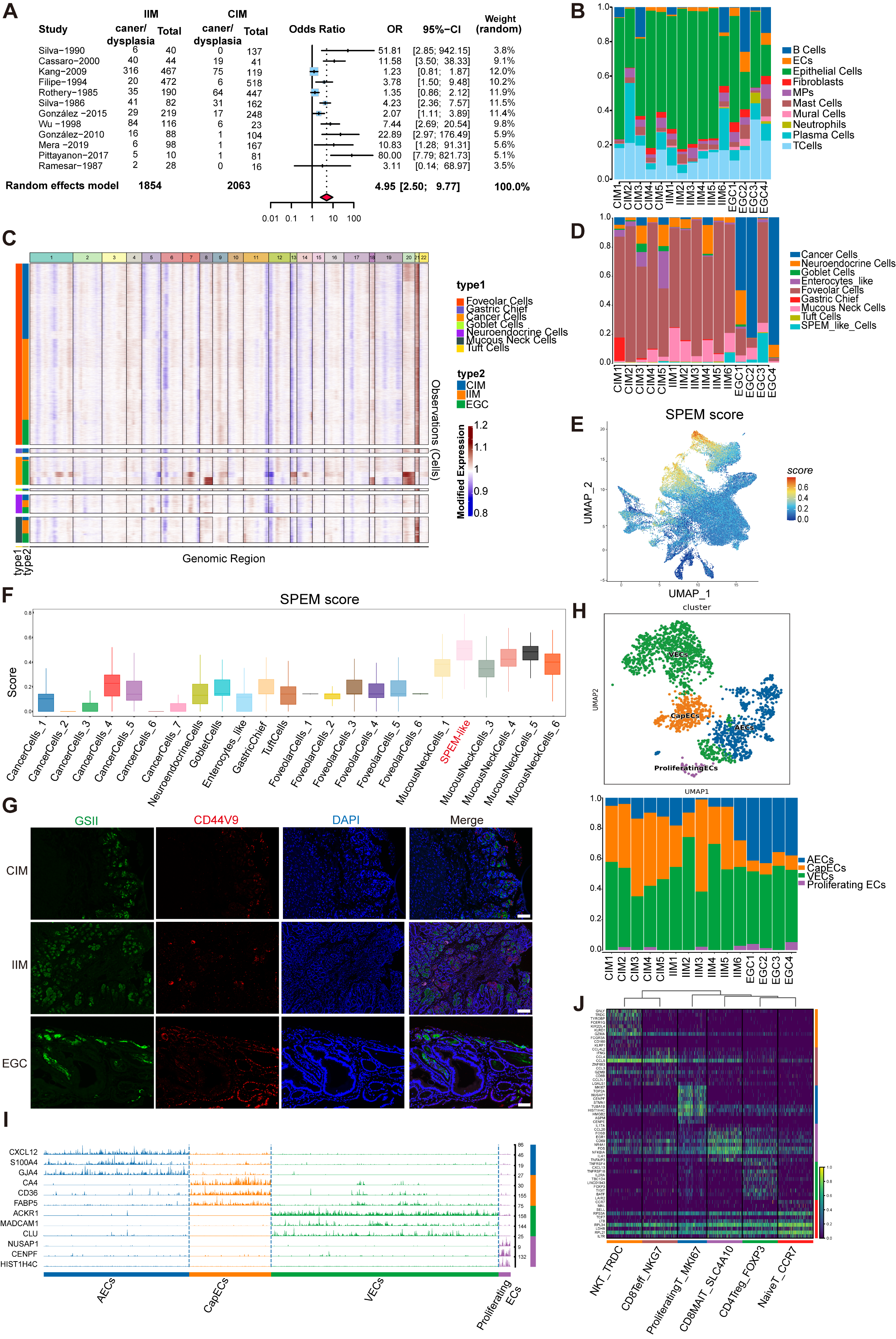
**

**Supplementary Figure1. Single-cell transcriptomic analysis reveals various cell types involved in intestinal-type gastric adenocarcinoma carcinogenesis, related to Figure1**

(A) Forest plot of the meta-analysis of the relative dysplasia or gastric cancer rates for IIM and CIM populations. (B) Fractions of major cell clusters detected in CIM, IIM and EGC groups. (C) CNV patterns of epithelial cell clusters in CIM, IIM and EGC samples. Rows correspond to cells. Red and blue colors indicate amplifications and deletions, respectively. (D) Fractions of epithelial cell clusters detected in CIM, IIM and EGC samples. (E) UMAP image showing the SPEM gene signature of epithelial cells. (F) Bar plots of the SPEM gene signature in epithelial cell clusters. (G) Representative mIHC images of the classic SPEM markers GSII and CD44v9 in CIM, IIM and EGC tissues. Scale bar, 50 μm. (H) UMAP of endothelial cell clusters colored according to inferred cell type (upper). Fractions of endothelial cell clusters detected in each group (bottom). (I) Tracksplot showing marker gene expression in endothelial cell clusters. (J) Heatmap of the DEGs (rows) corresponding to T-cell types (columns).

**
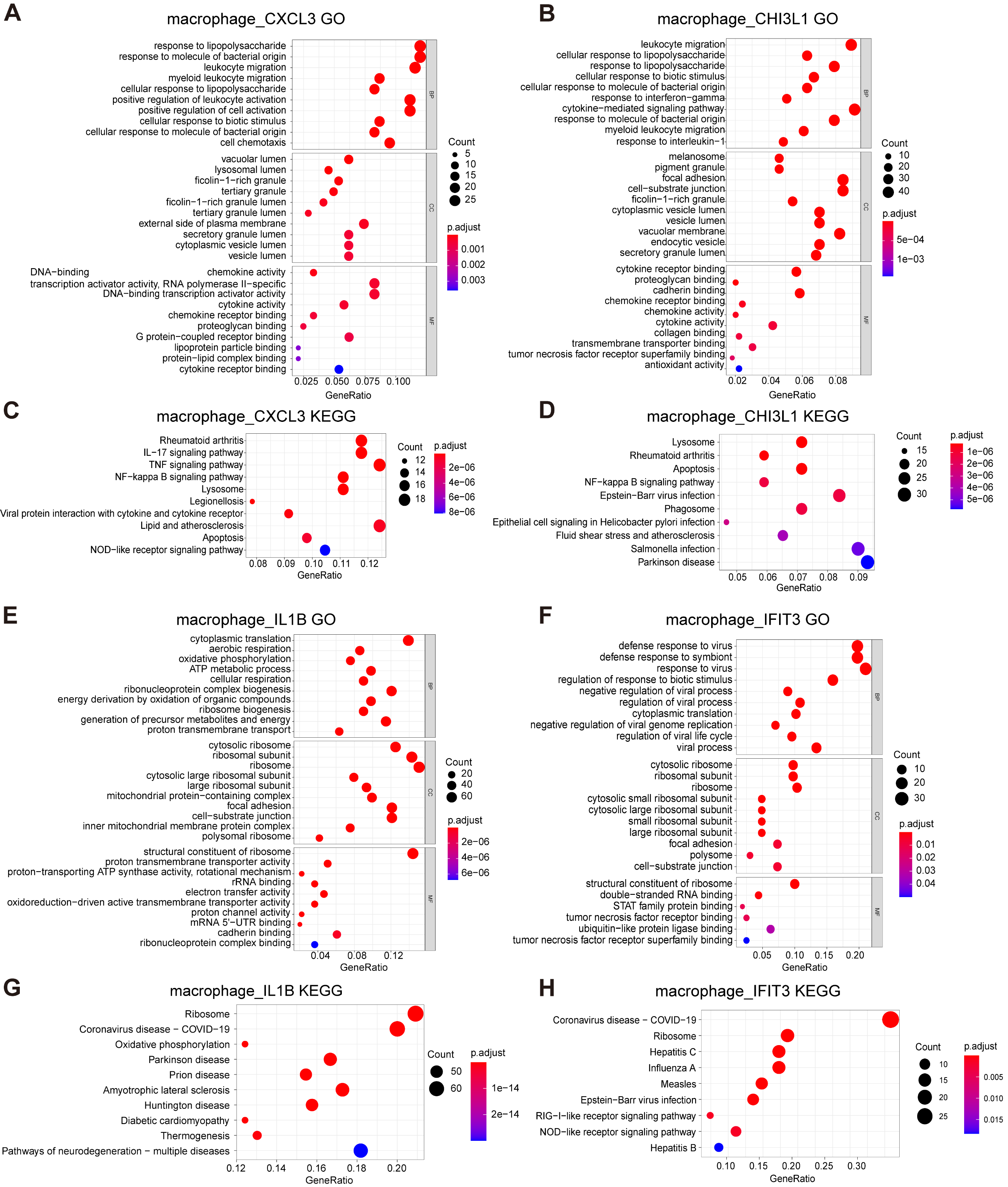
**

**Supplementary Figure2. Enrichment analysis of DEGs in five macrophage subtypes,** **related to Figure2**

(A, B, E, F) GO terms enriched with CXCL3^+^ (A), CHI3L1^+^ (B), IL-1B^+^ (E), and IFIT3^+^ (F) macrophages. (C, D, G, H) KEGG pathways enriched in CXCL3^+^ (C), CHI3L1^+^ (D), IL-1B^+^ (G), and IFIT3^+^ (H) macrophages. The color of the bubbles represents the adjusted P value, whereas the size of the bubbles represents the number of counts.

**
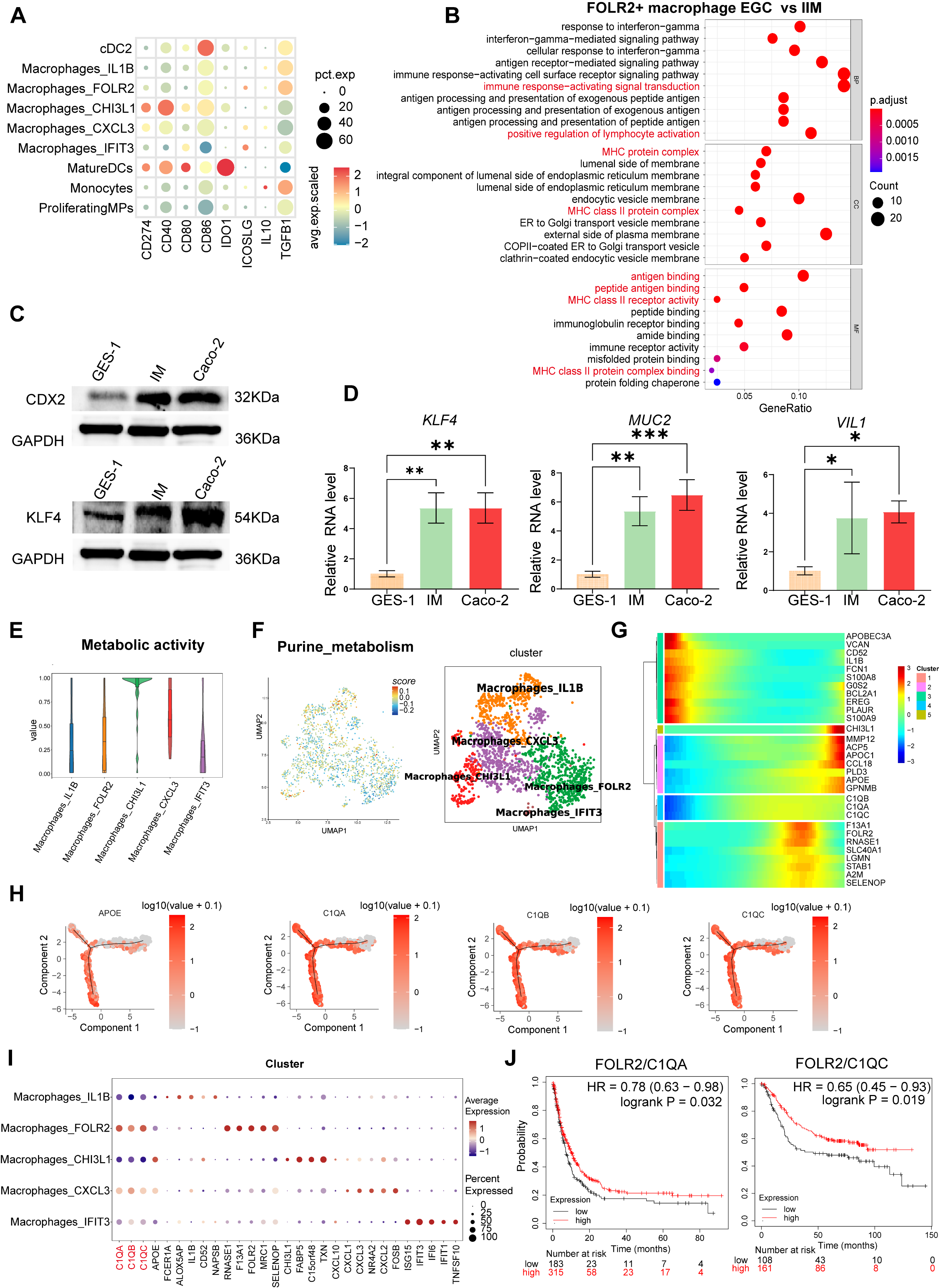
**

**Supplementary Figure3. FOLR2^+^ macrophages play crucial roles in antitumor immunity, related to Figure3**

(A) Bubble plot of immunosuppression-related genes in MP clusters. Bubble color denotes the average expression. The dot size indicates the proportion of expressing cells. (B) GO enrichment analysis of the upregulated DEGs in FOLR2^+^ macrophages from EGC compared with IIM tissues. The color gradient denotes adjusted P values. The dot size denotes the number of counts. (C) Western blot analysis of the levels of the intestinal markers CDX2 and KLF4 in GES-1, IM and Caco-2 cells. (D) RT‒qPCR analysis of *KLF4* (left), *MUC2* (middle), and *VIL* (right) mRNA levels in GES-1, IM and Caco-2 cells. (E) Violin plots showing the metabolic activity of macrophage clusters. (F) UMAP plot of the purine metabolism score in macrophage clusters. The color represents the metabolic value (left). UMAP plot of macrophage clusters (right) colored according to inferred cell type. (G) Heatmap of the expression of highly variable genes along the macrophage pseudotime trajectory. (H) Feature plots of the expression distributions of C1QA, C1QB, C1QC and APOE across pseudotime. (I) Bubble plot of the mean expression of specific genes in macrophage clusters. (J) The ratios of FOLR2/C1QA and FOLR2/C1QC were calculated to define the density of FOLR2^+^ macrophages. Kaplan‒Meier survival curves generated for the FOLR2^+^ macrophage density of TAMs from the KM plotter database. Patients were stratified into high- and low-ratio groups according to the optimal cutoff value. The data were analyzed by **the Kruskal‒Wallis test**. **P*<0.05, ***P*<0.01, ****P*<0.001; NS: not significant.

**
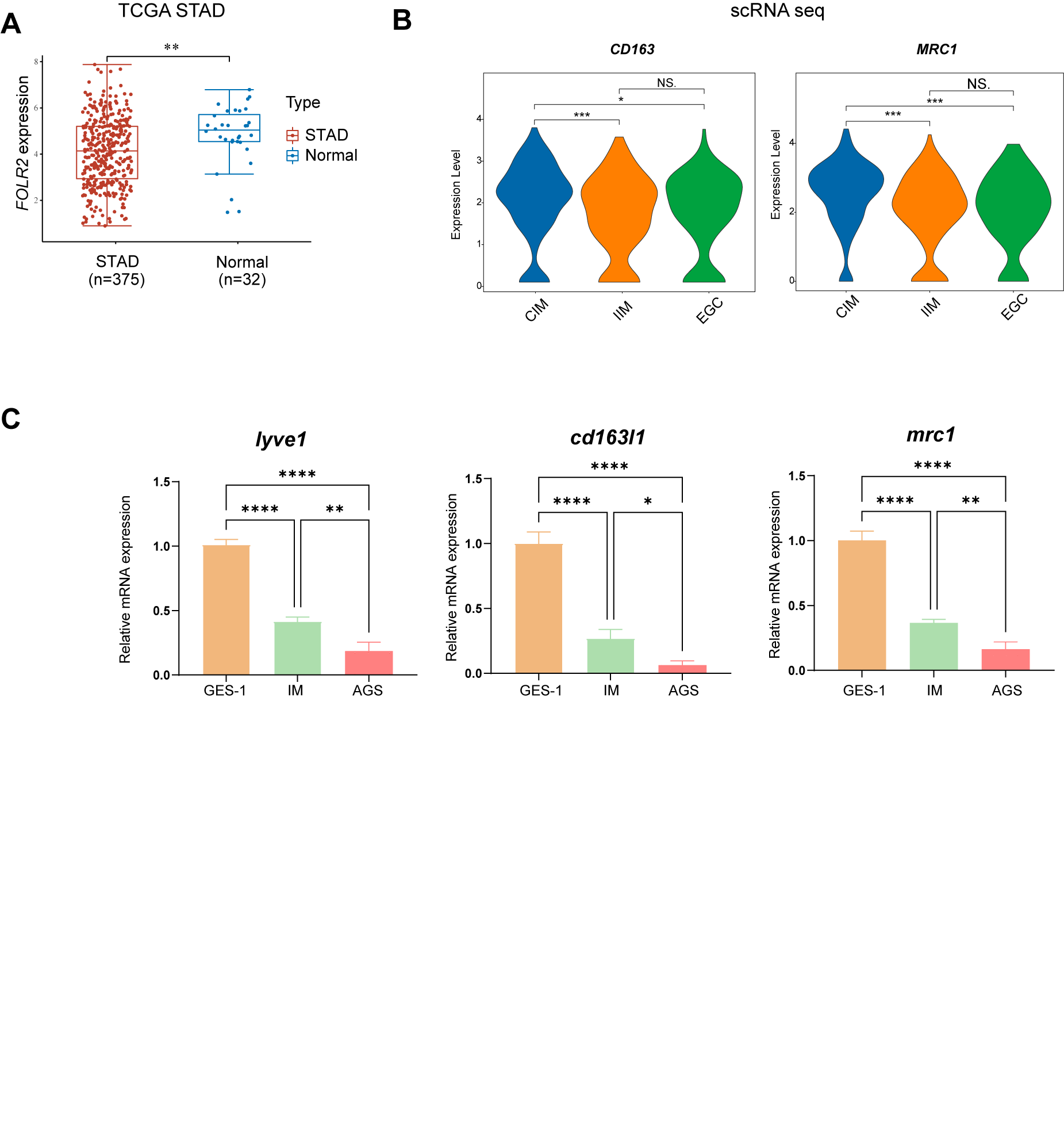
**

**Supplementary Figure4. FOLR2^+^ macrophages gradually decreased during EGC tumorigenesis，related to Figure4**

(A) The expression of FOLR2 in stomach cancer (STAD) and paracancerous normal (Normal) tissues from the TCGA database. (B) Violin plots of log-normalized *CD163 and MRC1* mRNA expression in FOLR2^+^ macrophages from our scRNA-seq data. (C) Log-normalized *LYVE1, CD163L1 and MRC1* mRNA expression in MDMs treated with GES-1, IM or AGS supernatants. The data were analyzed by the Wilcoxon rank-sum test or **the Kruskal‒Wallis test.** *p < 0.05; **p < 0.01; ***p < 10^-3^; ****p < 10^-4^; NS: not significant.

**
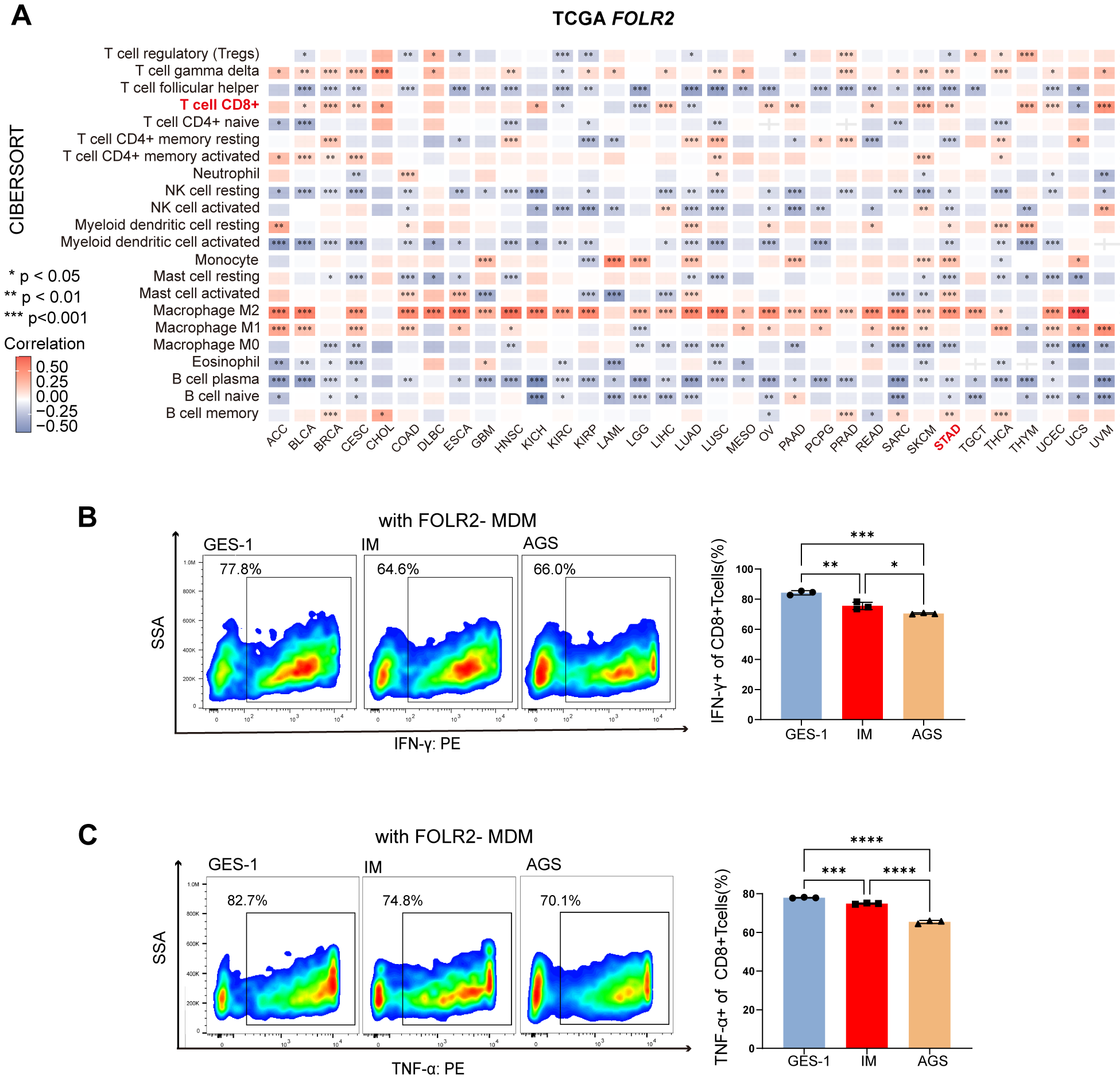
**

**Supplementary Figure5. FOLR2^+^ macrophages are positively correlated with CD8^+^ T cells during EGC carcinogenesis, related to Figure5**

(A) Spearman correlation between *FOLR2* expression and immune infiltration in TCGA pancancer atlas transcriptome data via CIBERSORT. The color gradient denotes the Spearman r coefficient. (B) Representative flow cytometry plots of IFN-γ expression in CD8^+^ T cells cocultured with FOLR2^-^ macrophages and different epithelial cell supernatants (n=3). (C) Representative flow cytometry plots of TNF-α expression in CD8^+^ T cells cocultured with FOLR2^-^ macrophages and different epithelial cell supernatants (n=3). The data were analyzed by Spearman correlation analysis or the Kruskal‒Wallis test. *p < 0.05; **p < 0.01; ***p < 10-3; ****p < 10-4; NS: not significant.

**
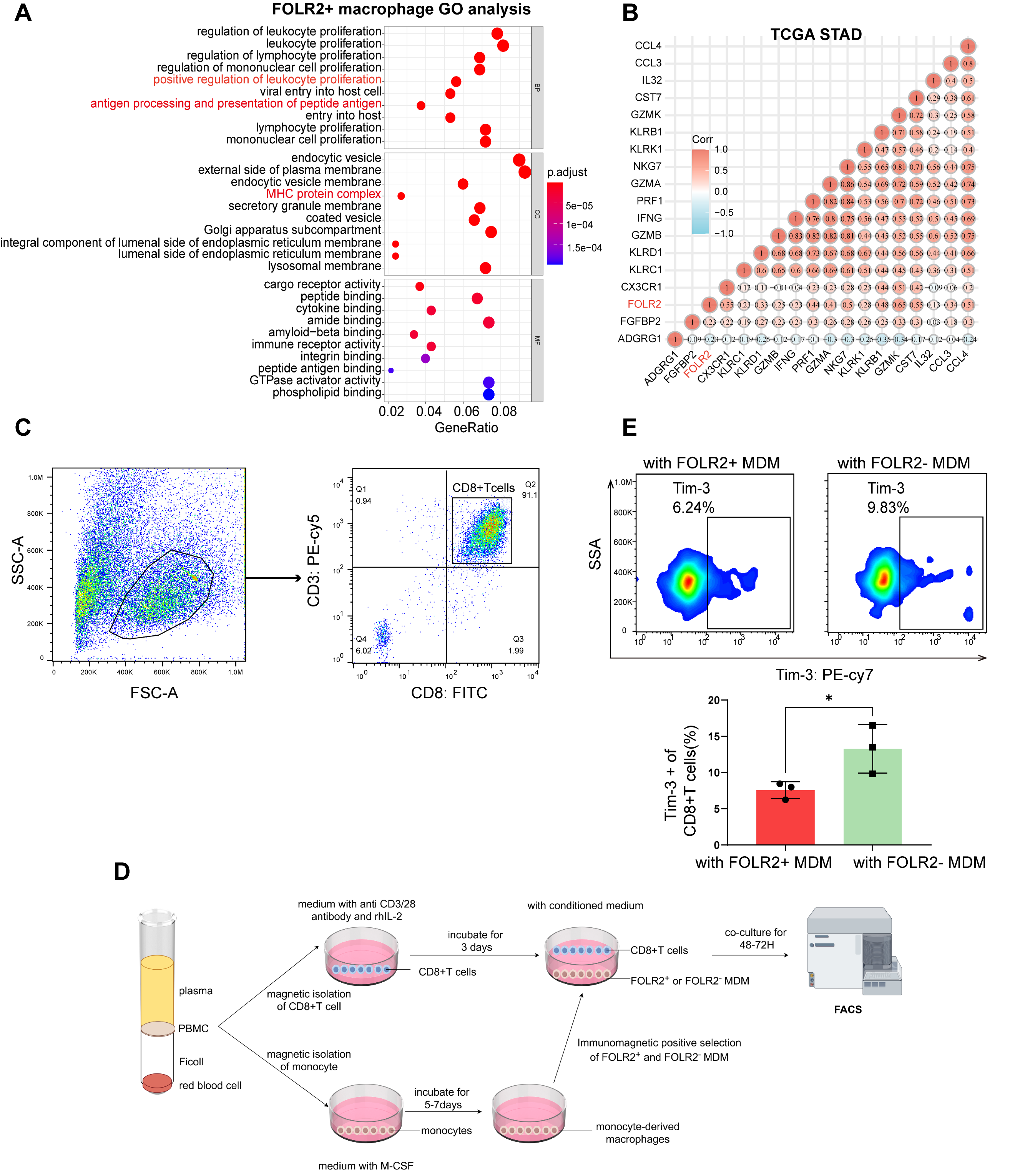
**

**Supplementary Figure6.** **FOLR2+ macrophages instruct CD8+ T-cell expansion and activation by antigen cross-presentation,** **related to Figure6**

(A) GO analysis of DEGs in FOLR2^+^ macrophages. (B) Spearman correlation between *FOLR2* expression and the cytotoxity-related genes of CD8^+^ Teffs in GC patients in the TCGA STAD database. The color gradient denotes the Spearman r coefficient. (C) Gating strategy for separating CD8^+^ T cells from the macrophage-CD8^+^ T cells coculture system. (D) Workflow of coculture experiments of autologous FOLR2^+^/^-^ macrophages and CD8^+^ T cells with epithelial cell supernatants. (E) Representative flow cytometry plots of Tim-3 expression in CD8^+^ T cells cocultured with FOLR2^+^ macrophages and FOLR2^-^ macrophages (n=3). The data were analyzed by Spearman correlation analysis or Student’s **t test.** *p < 0.05; **p < 0.01; ***p < 10^-3^; ****p < 10^-4^; NS: not significant.

**
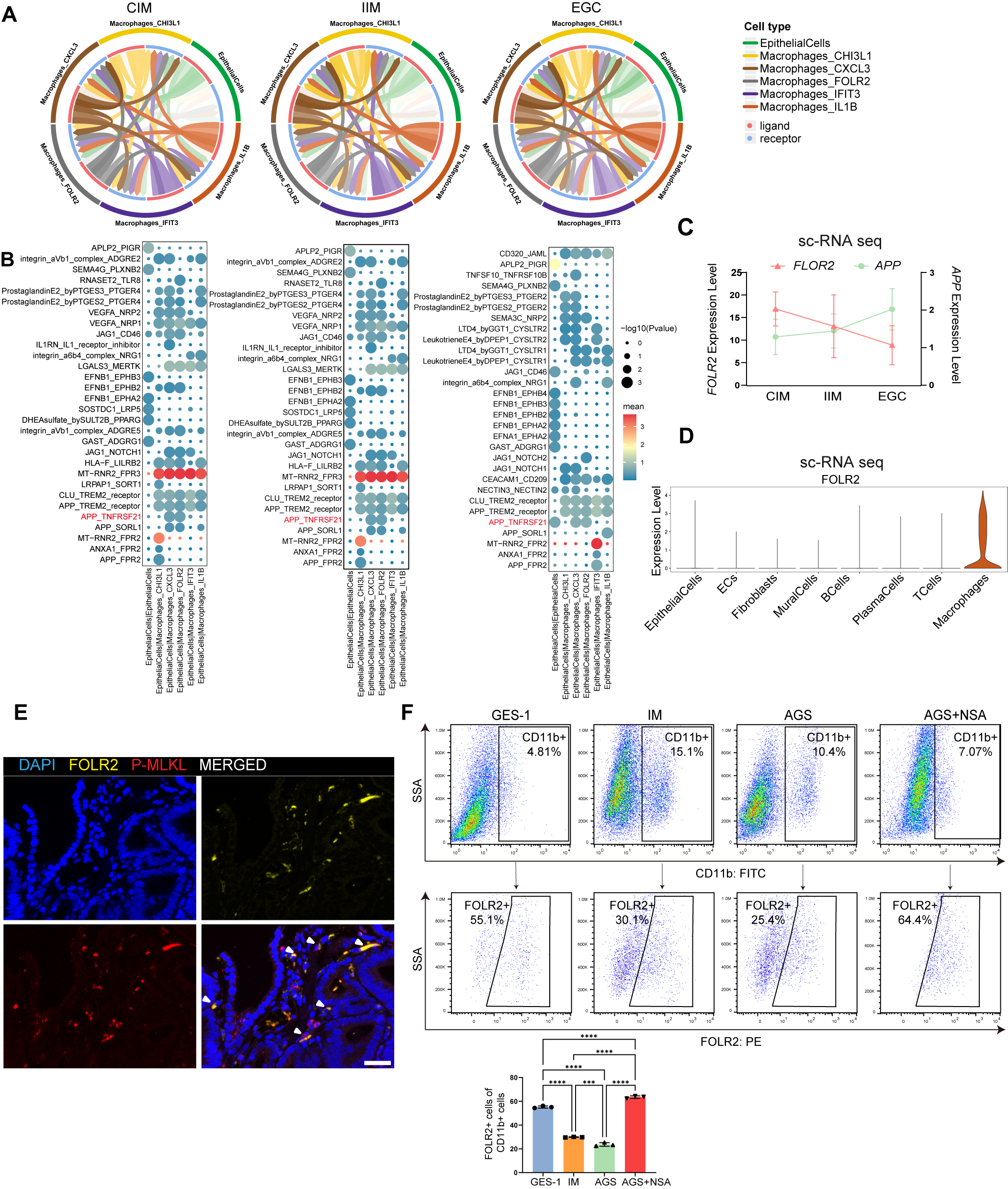
**

**Supplementary Figure7. APP upregulation in epithelial cells promotes necroptosis of FOLR2^+^ macrophages by enhancing the APP‒TNFRSF21 axis, related to Figure7**

(A) Chord diagrams of the interactions between epithelial cells and macrophage clusters. The outer color block represents the cell type. The inner ring is red for ligands and blue for receptors. Line clarity is positively correlated with the interaction number. (B) Bubble heatmap of the top 30 ligand‒receptor interactions between epithelial cell and macrophage clusters among CIMs, IIMs and EGCs via CellPhoneDB. The Y-axis represents ligand‒receptor pairs. The X-axis denotes the cell subsets. The color of the circle denotes the mean expression level. The p values were calculated with a one-sided permutation test. (C) Quantification of *FOLR2* mRNA levels in FOLR2^+^ macrophages and *APP* mRNA levels in epithelial cells among the different groups. (D) Violin plot of *FOLR2* mRNA levels in different cell types. (E) Representative mIHC images of FOLR2 and p-MLKL in EGC tissues. White arrows refer to FOLR2^+^ p-MLKL^+^ cells. Scale bar, 75 μm. (F) Representative flow cytometry plots of FOLR2 expression in CD11b^+^ MDMs cocultured with GES-1, IM, and AGS cells and NSA (n=3). The data were analyzed by **the Kruskal‒Wallis test.** *p < 0.05; **p < 0.01; ***p < 10^-3^; ****p < 10^-4^.
