# Supplementary material for: FOLR2+ macrophage depletion from intestinal metaplasia to early gastric cancer: single-cell sequencing insight into gastric cancer progression
Source: J Exp Clin Cancer Res. 2024 Dec 19;43:326. doi: 10.1186/s13046-024-03245-y (PMC11657096; doi:10.1186/s13046-024-03245-y)
Supplement: Supplementary file 2 — Supplementary Material 2 [file 13046_2024_3245_MOESM2_ESM.docx]

**Supplementary Methods**

**FOLR2^+^ macrophage depletion from intestinal metaplasia to early gastric cancer:**

**Single-cell sequencing insight into gastric cancer progression**

**Tissue Dissociation and Preparation**

Fresh tissues were stored in sCelLive™ Tissue Preservation Solution (Singleron Bio Com, Nanjing, China) on ice within 30 minutes post gastroscopy. The samples were washed three times with Hanks' balanced salt solution (HBSS) and then digested with 2 ml of sCelLive™ Tissue Dissociation Solution (Singleron) using a Singleron PythoN™ automated tissue dissociation system at 37 °C for 15 minutes. Subsequently, 2 ml of GEXSCOPE® red blood cell lysis buffer (Singleron) was added, and the cells were incubated at 25 °C for 10 minutes to remove the red blood cells. The mixture was then centrifuged at 500 × g for 5 minutes and gently resuspended in PBS. Finally, the samples were stained with trypan blue (Sigma, United States), and their viability was assessed microscopically.

**scRNA-seq Library Preparation**

Single-cell suspensions at a concentration of 1×10^5^ cells/ml in PBS were loaded into microfluidic devices using the Singleron Matrix® single-cell processing system (Singleron). The scRNA-seq libraries were then constructed following the protocol of the GEXSCOPE® single-cell RNA library kit (Singleron) [1]. Individual libraries were diluted to 4 nM and pooled for sequencing. The pooled libraries were sequenced on an Illumina NovaSeq 6000 platform with 150 bp paired-end reads.

**Preprocessing of scRNA-seq Data**

For the data generated using the Singleron kits, the reads were aligned to the human reference genome (GRCh38), and unique molecular identifier (UMI) counts were obtained using the featureCounts software. To filter out low-quality, dead, or stressed cells, the following thresholds were applied: Cells with fewer than 50 genes per cell, cells with more than 30,000 UMIs per cell, and cells with more than 20% of counts derived from mitochondrial genes. After quality control, the filtered gene expression matrix was used for downstream analysis.

**Quality Control, Dimension Reduction, and Clustering (Scanpy)**

Scanpy v1.8.1 [2] (RRID:SCR_018139) was used for quality control, dimensionality reduction, and clustering with Python 3.7. For each sample dataset, the expression matrix was filtered using the following criteria: (1) Cells with fewer than 200 genes or within the top 2% of gene counts were excluded. (2) Cells within the top 2% of UMI counts were excluded. (3) Cells with a mitochondrial content greater than 50% were excluded. (4) Genes expressed in fewer than 5 cells were excluded. After filtering, 96,125 cells were retained for downstream analyses, with an average of 1,057.946 genes and 3,647.05 UMIs per cell. The raw count matrix was normalized by total counts per cell and logarithmically transformed into a normalized data matrix. The top 2,000 variable genes were selected by setting flavor='seurat'. Principal component analysis (PCA) was performed on the scaled variable gene matrix, and the top 20 principal components were used for clustering and dimensionality reduction. The cells were separated into 22 clusters using the Louvain algorithm with the resolution parameter set to 1.2. The cell clusters were visualized via uniform manifold approximation and projection (UMAP).

**Cell Type Annotation**

We identified the top differential markers for each cluster and subcluster via the “FindAllMarkers” function from the “Seurat” R package (RRID:SCR_007322). For each cell type, we leveraged a combination of cell type-specific and enriched marker genes. The marker genes used for annotating different cell subpopulations are listed in Supplementary Table 1.

**Differentially Expressed Genes (DEGs) Analysis (Scanpy)**

To identify differentially expressed genes (DEGs), we used the “scanpy.tl.rank_genes_groups” function based on the Wilcoxon rank sum test with default parameters. Genes were considered DEGs if they were expressed in more than 10% of the cells in either of the compared groups and had an average log (fold change) value greater than 0.25. The adjusted p value was calculated using the Benjamini‒Hochberg correction, with a threshold of 0.05 for statistical significance.

**scRNA-seq-Based CNA Detection**

The InferCNV package [3] (RRID:SCR_021140) was used to detect copy number alterations (CNAs) in malignant epithelial cells. Nonmalignant cells were used as baselines to estimate the CNAs of the malignant cells. Genes expressed in more than 20 cells were sorted on the basis of their loci on each chromosome. The relative expression values were centered to 1, using 1.5 standard deviations from the residual-normalized expression values as the floor and ceiling. A sliding window size of 101 genes was used to smooth the relative expression on each chromosome, removing the effect of gene-specific expression. The inferred CNAs on each short or long arm or the full length of the chromosomes were visualized with heatmaps generated by the R pheatmap function. The clonal relationships of the malignant cell clusters in each sample were determined by the accumulation of CNAs. UPhyloplot2 software was used to draw clonality trees of malignant cell clusters, with CNA events manually labeled on the branches. The CNV score of each cell was calculated as the quadratic sum of the CNA regions.

For each cell, the CNV score is defined as the average of the absolute values of the inferred CNVs, obtained by subtracting 1 from the CNV values in the matrix output by InferCNV (RRID:SCR_021140).

**Gene Pathway Enrichment Analysis**

To investigate potential functions, Gene Ontology (GO) and Kyoto Encyclopedia of Genes and Genomes (KEGG) analyses were performed using the “clusterProfiler” R package v4.0 (RRID:SCR_016884) [4,5]. Pathways with an adjusted p value (p_adj) less than 0.05 were considered significantly enriched. Selected significant pathways are visualized as bar plots. For gene set variation analysis (GSVA) pathway enrichment analysis, the average gene expression of each cell type was used as the input data [6]. Gene Ontology gene sets, including molecular function (MF), biological process (BP), and cellular component (CC) categories, were utilized.

**Gene Signature Scores Based on scRNA-seq Data**

The macrophage function signature and SPEM score were calculated using the R package “UCell” v2.2.0. All of the gene sets involved in the calculation are listed in Supplementary Table 3.

**Pseudotime Trajectory Analysis: Monocle2**

The cell differentiation trajectories of monocyte subtypes were reconstructed using “Monocle2” v2.22.0 (RRID:SCR_016339) [7]. To construct the trajectory, the top 2000 highly variable genes were selected using R package “Seurat” (v3.1.2) “FindVariableFeatures”, and dimension reduction was performed using “DDRTree”. The trajectory was visualized using the “plot_cell_trajectory” function in the Monocle2 R package.

**Transcription Factor Regulon by SCENIC**

Transcription factor networks were constructed using pySCENIC (RRID:SCR_025802) (v0.11.0) [8] with the scRNA expression matrix and transcription factors from AnimalTFDB. First, GRNBoost2 predicted a regulatory network on the basis of the coexpression of regulators and targets. CisTarget was then applied to exclude indirect targets and search for transcription factor binding motifs. Subsequently, AUCell was used for regulon activity quantification for each cell. Cluster-specific transcription factor (TF) regulons were identified according to Regulon specificity scores (RSSs), and the activity of these TF regulons was visualized using heatmaps.

**Cell‒cell interaction analysis: NicheNet**

The cell‒cell interaction (CCI) between FOLR2^+^ macrophages and CD8^+^ T cells was predicted on the basis of known ligand‒receptor pairs by NicheNet (RRID:SCR_023158) in R [9]. Cell type signatures were derived from the top 100 DEGs from FOLR2^+^ macrophages and CD8^+^ T cells. Using DEGs, cell type-specific ligands were predicted and ranked by regulatory potential. The top 15% of the predicted ligands were considered significant and visualized in heatmaps.

**Cell‒cell interaction analysis: CellPhoneDB**

The CCI between epithelial cells and FOLR2^+^ macrophages was predicted on the basis of known ligand–receptor pairs using CellPhoneDB (RRID:SCR_017054) (v4.0.0) [10]. The permutation number for calculating the null distribution of average ligand‒receptor pair expression with randomized cell identities was set to 1000. Individual ligand or receptor expression was thresholded by a cutoff on the basis of the average log gene expression distribution for all genes across each cell type. Predicted interaction pairs with a p value <0.05 and an average log expression >0.1 were considered significant and visualized using the “heatmap_plot” and “dot_plot” functions in CellPhoneDB.

**Single-Cell Metabolism Analysis: scMetabolism**

scMetabolism (v0.2.1), an R package designed for the quantification of single-cell metabolic activity, was applied for analysis [11]. KEGG metabolic pathway data were collected, and the metabolic pathway enrichment scores were calculated on the basis of VISION. The scores of specific pathways were visualized using the FeaturePlot and VlnPlot functions in Seurat.

**KM plotter data**

The correlation between overall survival (OS) and gene expression in gastric cancer (GC) patients was obtained from the Kaplan‒Meier plotter gastric cancer dataset ([www.kmplot.com](http://www.kmplot.com)) (RRID:SCR_024521) [12]. We used FOLR2/C1QA and FOLR2/C1QC to calculate the survival probability of GC patients.

**TCGA Data and Analysis**

Bulk RNA-sequencing expression profiles and corresponding clinical information for GC patients were downloaded from the TCGA dataset (RRID:SCR_003193) (https://portal.gdc.com). Spearman’s correlation analysis was performed to determine the correlation between *FOLR2* and other genes. The two-gene correlation analysis scatter plot was created using the R package “ggstatsplot”, whereas multigene correlations were visualized using a correlation diagonal heatmap created with the R package corrplot (RRID:SCR_024683).

**CIBERSORT Immune Infiltration Estimation**

The associations between immune infiltrates and gene expression were obtained from TIMER 2.0 (RRID:SCR_018737) (<http://timer.cistrome.org/>) [13]. We investigated the relationship between *FOLR2* gene expression and immune infiltrates across diverse cancer types.

**Reference**

1. Dura B, Choi JY, Zhang K, et al. scFTD-seq: freeze-thaw lysis based, portable approach toward highly distributed single-cell 3' mRNA profiling. *Nucleic Acids Res* 2019;47:e16.

2. Wolf FA, Angerer P, Theis FJ. SCANPY: large-scale single-cell gene expression data analysis. *Genome Biol* **2018**;19:15.

3. Tirosh I, Venteicher AS, Hebert C, et al. Single-cell RNA-seq supports a developmental hierarchy in human oligodendroglioma. *Nature* **2016**;539:309-313.

4. Yu G, Wang LG, Han Y, et al. clusterProfiler: an R package for comparing biological themes among gene clusters. *OMICS* **2012**;16:284-7.

5. Liberzon A, Birger C, Thorvaldsdottir H, et al. The Molecular Signatures Database (MSigDB) hallmark gene set collection. *Cell Syst* **2015**;1:417-425.

6. Hanzelmann S, Castelo R, Guinney J. GSVA: gene set variation analysis for microarray and RNA-seq data. *BMC Bioinformatics* **2013**;14:7.

7. Andreatta M, Carmona SJ. UCell: Robust and scalable single-cell gene signature scoring. *Comput Struct Biotechnol J* **2021**;19:3796-3798.

8. Van de Sande B, Flerin C, Davie K, et al. A scalable SCENIC workflow for single-cell gene regulatory network analysis. *Nat Protoc* **2020**;15:2247-2276.

9. Browaeys R, Saelens W, Saeys Y. NicheNet: modeling intercellular communication by linking ligands to target genes. *Nat Methods* **2020**;17:159-162.

10. Efremova M, Vento-Tormo M, Teichmann SA, et al. CellPhoneDB: inferring cell-cell communication from combined expression of multi-subunit ligand-receptor complexes. *Nat Protoc* **2020**;15:1484-1506.

11. Wu Y, Yang S, Ma J, et al. Spatiotemporal Immune Landscape of Colorectal Cancer Liver Metastasis at Single-Cell Level. *Cancer Discov* **2022**;12:134-153.

12. Gyorffy B. Integrated analysis of public datasets for the discovery and validation of survival-associated genes in solid tumors. *Innovation (Camb)* **2024**;5:100625.

13. Li T, Fu J, Zeng Z, et al. TIMER2.0 for analysis of tumor-infiltrating immune cells. *Nucleic Acids Res* **2020**;48:W509-W514.
